# On the overestimation of random forest’s out-of-bag error

**DOI:** 10.1371/journal.pone.0201904

**Published:** 2018-08-06

**Authors:** Silke Janitza, Roman Hornung

**Affiliations:** Institute for Medical Information Processing, Biometry and Epidemiology, University of Munich, Munich, Germany; Chuo University, JAPAN

## Abstract

The ensemble method random forests has become a popular classification tool in bioinformatics and related fields. The out-of-bag error is an error estimation technique often used to evaluate the accuracy of a random forest and to select appropriate values for tuning parameters, such as the number of candidate predictors that are randomly drawn for a split, referred to as *mtry*. However, for binary classification problems with metric predictors it has been shown that the out-of-bag error can overestimate the true prediction error depending on the choices of random forests parameters. Based on simulated and real data this paper aims to identify settings for which this overestimation is likely. It is, moreover, questionable whether the out-of-bag error can be used in classification tasks for selecting tuning parameters like *mtry*, because the overestimation is seen to depend on the parameter *mtry*. The simulation-based and real-data based studies with metric predictor variables performed in this paper show that the overestimation is largest in balanced settings and in settings with few observations, a large number of predictor variables, small correlations between predictors and weak effects. There was hardly any impact of the overestimation on tuning parameter selection. However, although the prediction performance of random forests was not substantially affected when using the out-of-bag error for tuning parameter selection in the present studies, one cannot be sure that this applies to all future data. For settings with metric predictor variables it is therefore strongly recommended to use stratified subsampling with sampling fractions that are proportional to the class sizes for both tuning parameter selection and error estimation in random forests. This yielded less biased estimates of the true prediction error. In unbalanced settings, in which there is a strong interest in predicting observations from the smaller classes well, sampling the same number of observations from each class is a promising alternative.

## Introduction

Random forests (RF) [1] have become a popular classification tool in bioinformatics and related fields. They have also shown excellent performance in very complex data settings. Each tree in a RF is constructed based on a random sample of the observations, usually a bootstrap sample or a subsample of the original data. The observations that are not part of the bootstrap sample or subsample, respectively, are referred to as out-of-bag (OOB) observations. The OOB observations can be used for example for estimating the prediction error of RF, yielding the so-called OOB error. The OOB error is often used for assessing the prediction performance of RF. An advantage of the OOB error is that the complete original sample is used both for constructing the RF classifier and for error estimation. By contrast, with cross-validation and related data splitting procedures for error estimation a subset of the samples are left out for RF construction, which is why the resulting RF classifiers are less performant. Another advantage of using the OOB error is its computational speed. In contrast to cross-validation or other data splitting approaches, only one RF has to be constructed, while for *k*-fold cross-validation *k* RF have to be constructed [2, 3]. The use of the OOB error saves memory and computation time, especially when dealing with large data dimensions, where constructing a single RF might last several days or even weeks. These reasons might explain the frequent use of the OOB error for error estimation and tuning parameter selection in RF.

The OOB error is often claimed to be an unbiased estimator for the true error rate [1, 3, 4]. However, for two-class classification problems it was reported that the OOB error can overestimate the true prediction error depending on the choices of RF parameters [2, 5]. The bias can be very substantial, as shown in the latter papers, and is also present when using classical cross-validation procedures for error estimation. It was thus recommended by Mitchell [5] to use the OOB error only as an upper bound for the true prediction error. However, Mitchell [5] considered only settings with completely balanced samples, sample sizes below 60 and two response classes, limiting the generality of his results.

Besides the fact that trees in RF are constructed on a random sample of the data, there is a second component which differs between standard classification and regression trees and the trees in RF. In the trees of a RF, not all variables but only a subset of the variables are considered for each split. This subset is randomly drawn from all candidate predictors at each split. The size of this subset is usually referred to as *mtry*. The purpose of considering random subsets of the predictors instead of all predictors is to reduce the correlation between the trees, that is, to make them more dissimilar. This reduces the variance of the predictions obtained using RF. In practical applications, the most common approach for choosing appropriate values for *mtry* is to select the value over a grid of plausible values that yields the smallest OOB error [6–8]. In literature on RF methodology, the OOB error has also frequently been used to choose an appropriate value for *mtry* [9, 10]. In principle, other procedures like (repeated) cross-validation may be applied for selecting an optimal value for *mtry*, but the OOB error is usually the first choice for parameter tuning. This is due to the fact that, unlike many other approaches such as cross-validation, the whole data can be used to construct the RF and much computational effort is saved since only one RF has to be built for each candidate *mtry* value. Implementations exist that use the OOB error to select an appropriate value for *mtry*. In the statistical software R [11], for example, the function tuneRF from the package randomForest [12] automatically searches over a grid of *mtry* values and selects the value for *mtry* for which the OOB error is smallest. The latter approach is only valid, if the bias of the OOB error does not depend on the parameter *mtry*. If, by contrast, the bias of the OOB error does depend on this parameter, the *mtry* value minimizing the OOB error cannot be expected to minimize the true prediction error and will thus be in general suboptimal, making the OOB error based tuning approaches questionable. To date there are no studies investigating the reliability of the OOB error for tuning parameters like *mtry* in RF.

The main contribution of this paper is three-fold: (i) the bias and its dependence on *mtry* in settings with metric predictor variables are quantitatively assessed through studies with different numbers of observations, predictors and response classes which helps to identify so-called “high-risk settings”, (ii) the reasons for this bias and its dependence on *mtry* are studied in detail, and based on these findings, the use of alternatives, such as stratified sampling (which preserves the response class distribution of the original data in each subsample), is investigated, and (iii) the consequences of the bias for tuning parameter selection are explored.

This paper is structured as follows: In the section “Methods”, simulation-based and real-data based studies are described after briefly introducing the RF method. The description includes an outline of the simulated and real data, the considered settings and several different error estimation techniques that will be used. The results of the studies are subsequently shown in the section “Results”, where also some additional studies are presented, and finally recommendations are given. The results are discussed in the section “Discussion” and the main points are condensed in the section “Conclusions”.

## Methods

In this section, the RF method and the simulation-based and real-data based studies are described. Simulated data is used to study the behavior of the OOB error in simple settings, in which all predictors are uncorrelated. This provides insight to the mechanisms which lead to the bias in the OOB error. Based on these results, settings are identified, in which a bias in the OOB error is likely. Subsequently, to assess the extent of the bias in these settings in practice, complex real world data is used.

### Random forests and its out-of-bag error

RF is an ensemble of classification or regression trees that was introduced by Breiman [1]. One of the two random components in RF concerns the choice of variables used for splitting. For each split in a tree, the best splitting variable from a random sample of *mtry* predictors is selected. If the chosen *mtry* value is too small, it might be that none of the variables contained in the subset is relevant and that irrelevant variables are often selected for a split. The resulting trees have poor predictive ability. If the subset contains a large number of predictors, in contrast, it is likely that the same variables, namely those with the largest effect, are often selected for a split and that variables with smaller effects have hardly any chance of being selected. Therefore, *mtry* should be considered a tuning parameter.

The other random component in RF concerns the choice of training observations for a tree. Each tree in RF is built from a random sample of the data. This is usually a bootstrap sample or a subsample of size 0.632*n*. Therefore not all observations are used to construct a specific tree. The observations that are not used to construct a tree are denoted by *out-of-bag (OOB) observations*. In a RF, each tree is built from a different sample of the original data, so each observation is “out-of-bag” for some of the trees. The prediction for an observation can then be obtained by using only those trees for which the observation was not used for the construction. A classification for each observation is obtained in this way and the error rate can be estimated from these predictions. The resulting error rate is referred to as *OOB error*. This procedure was originally introduced by Breiman [13] and it has become an established method for error estimation in RF.

### Simulation-based studies

The upward bias of the OOB error in different data settings with metric predictor variables was systematically investigated by means of simulation studies.

Settings were considered with

- different associations between the predictors and the response. Either none of the predictors were associated with the response (the corresponding studies termed *null case*) or some of them were associated (*power case*);
- different numbers of predictors, *p* ∈ {10, 100, 1000};
- different numbers of response classes, *k* ∈ {2, 4}. The studies are termed *binary* if *k* = 2 and *multiclass* if *k* = 4;
- different response class ratios. An equal number of observations of each response class was used (*balanced settings*) for *k* ∈ {2, 4}. For *k* = 2 two additional settings with unequal response class sizes were simulated (*binary unbalanced* and *binary extremely unbalanced*). In the first setting (*binary unbalanced*), the smaller class comprised 30% of the observations. In the second setting (*binary extremely unbalanced*), the smaller class comprised approximately 17% (ratio 1:5) of the observations.
- different numbers of observations, *n* ∈ {*n*_*small*_, 100, 1000}, with *n*_*small*_ = 20 for *binary balanced* studies, *n*_*small*_ = 30 for *binary unbalanced* studies, *n*_*small*_ = 60 for *binary extremely unbalanced* studies and *n*_*small*_ = 40 for *multiclass balanced* studies.

Since one of the aims was to investigate the bias in dependence on *mtry*, several RFs with different *mtry* values were constructed for each setting. The grid of considered *mtry* values was {1, 2, 3, …, 10} for *p* = 10, {1, 10, 20, 30, …, 100} for *p* = 100 and {1, 5, 10, 50, 100, 200, 300, …, 1000} for *p* = 1000. Note that for *mtry* = 1 there is no selection of an optimal predictor variable for a split, while for *mtry* = *p* the RF method coincides with the bagging procedure which selects the best predictor variable from all available predictors [14]. A large number of trees, referred to as *ntree*, should be chosen especially if the data are composed of a large number of predictors. It is usually chosen by considering a compromise between accuracy and computational speed. The OOB error stabilized at around 250 trees in convergence studies of Goldstein et al. [15], and they concluded that 1000 trees might be sufficiently large for their genome-wide data set. Also in the studies of Díaz-Uriarte and De Andres [16] the results for RF with 1000 trees were almost the same as those for RF with 40000 trees, and in the high-dimensional settings of Genuer et al. [17] RF with 500 trees and 1000 trees yielded very similar OOB errors. In accordance with these findings the number of trees was set to 1000 in all studies of this paper (including at most ∼ 7000 predictors). Each setting was repeated 500 times to obtain stable results.

Only metric predictor variables were considered in the studies. In the null case study, the predictors *X*_1_, …, *X*_*p*_ were independent and identically distributed (*i.i.d.*), each following a standard normal distribution (see Tables 1 and 2). In the power case study, both, predictors associated with the response and predictors not associated with the response were considered. The predictors not associated with the response followed a standard normal distribution. The distribution of predictors with association was different for each response class. The predictor values for observations from class 1 were always drawn from a standard normal distribution. The predictor values for observations from class 2 (or classes 2, 3, and 4 in settings with *k* = 4 response classes) were drawn from a normal distribution with variance 1 and a mean different from zero. Tables 1 and 2 give an overview of the distribution of predictors in the response classes for settings with *k* = 2 and *k* = 4 response classes, respectively. Let us consider the setting with *p* = 10 and *k* = 4 as an example (Table 2). The first two predictors *X*_1_ and *X*_2_ are associated with the response, while the other predictors *X*_3_, …, *X*_10_ are not. Accordingly, *X*_3_, …, *X*_10_ always follow a standard normal distribution, while the distribution of *X*_1_ and *X*_2_ depends on whether the observation comes from class 1 or from a different class. If the observation comes from class 1 the distribution of *X*_1_ and *X*_2_ is *N*(0, 1), and if it comes from class *r* ∈ {2, 3, 4} the variables *X*_*j*_, *j* = 1, 2 follow a normal distribution *N*(*μ*_*rj*_, 1) with *μ*_*rj*_ drawn independently from *N*(0.4, 1). Randomly drawing the mean separately for *X*_1_ and *X*_2_ and for each repetition of the study makes sure that predictors with different effect strengths are considered.

**Table 1:** Simulation design: Two response classes.

Study	No. predictors	Predictors	Class 1 *N*(*μ*_1_, 1)	Class 2 *N*(*μ*_2_, 1)
*Null case*	*p* ∈ {10, 100, 1000}	*X*_1_, …, *X*_*p*_	*μ*_1_ = 0	*μ*_2_ = 0
*Power case*	*p* = 10	*X*_1_	*μ*_1_ = 0	*μ*_2_ ∼ *N*(0.75, 1)
		*X*_2_	*μ*_1_ = 0	*μ*_2_ ∼ *N*(0.75, 1)
		*X*_3_, …, *X*_10_	*μ*_1_ = 0	*μ*_2_ = 0
	*p* = 100	*X*_1_	*μ*_1_ = 0	*μ*_2_ ∼ *N*(0.75, 1)
		⋮	⋮	⋮
		*X*_10_	*μ*_1_ = 0	*μ*_2_ ∼ *N*(0.75, 1)
		*X*_11_, …, *X*_100_	*μ*_1_ = 0	*μ*_2_ = 0
	*p* = 1000	*X*_1_	*μ*_1_ = 0	*μ*_2_ ∼ *N*(0.1, 1)
		⋮	⋮	⋮
		*X*_50_	*μ*_1_ = 0	*μ*_2_ ∼ *N*(0.1, 1)
		*X*_51_, …, *X*_1000_	*μ*_1_ = 0	*μ*_2_ = 0

Distribution of predictors in class 1 and class 2 of the simulated data setting with *k* = 2 response classes.

**Table 2:** Simulation design: Four response classes.

Study	No. predictors	Predictors	Class 1 *N*(*μ*_1_, 1)	Class 2 *N*(*μ*_2_, 1)	Class 3 *N*(*μ*_3_, 1)	Class 4 *N*(*μ*_4_, 1)
*Null case*	*p* ∈ {10, 100, 1000}	*X*_1_, …, *X*_*p*_	*μ*_1_ = 0	*μ*_2_ = 0	*μ*_3_ = 0	*μ*_4_ = 0
*Power case*	*p* = 10	*X*_1_	*μ*_1_ = 0	*μ*_2_ ∼ *N*(0.4, 1)	*μ*_3_ ∼ *N*(0.4, 1)	*μ*_4_ ∼ *N*(0.4, 1)
		*X*_2_	*μ*_1_ = 0	*μ*_2_ ∼ *N*(0.4, 1)	*μ*_3_ ∼ *N*(0.4, 1)	*μ*_4_ ∼ *N*(0.4, 1)
		*X*_3_, …, *X*_10_	*μ*_1_ = 0	*μ*_2_ = 0	*μ*_3_ = 0	*μ*_4_ = 0
	*p* = 100	*X*_1_	*μ*_1_ = 0	*μ*_2_ ∼ *N*(0.4, 1)	*μ*_3_ ∼ *N*(0.4, 1)	*μ*_4_ ∼ *N*(0.4, 1)
		⋮	⋮	⋮	⋮	⋮
		*X*_10_	*μ*_1_ = 0	*μ*_2_ ∼ *N*(0.4, 1)	*μ*_3_ ∼ *N*(0.4, 1)	*μ*_4_ ∼ *N*(0.4, 1)
		*X*_11_, …, *X*_100_	*μ*_1_ = 0	*μ*_2_ = 0	*μ*_3_ = 0	*μ*_4_ = 0
	*p* = 1000	*X*_1_	*μ*_1_ = 0	*μ*_2_ ∼ *N*(0.4, 1)	*μ*_3_ ∼ *N*(0.4, 1)	*μ*_4_ ∼ *N*(0.4, 1)
		⋮	⋮	⋮	⋮	⋮
		*X*_50_	*μ*_1_ = 0	*μ*_2_ ∼ *N*(0.4, 1)	*μ*_3_ ∼ *N*(0.4, 1)	*μ*_4_ ∼ *N*(0.4, 1)
		*X*_51_, …, *X*_1000_	*μ*_1_ = 0	*μ*_2_ = 0	*μ*_3_ = 0	*μ*_4_ = 0

Distribution of predictors in class 1, class 2, class 3 and class 4 of the simulated data setting with *k* = 4 response classes.

Despite considering metric predictors with different effect strengths, the settings are simplistic because all predictors are uncorrelated. Although assuming no correlations between any of the predictors is not realistic, such settings are important to understand the mechanisms which lead to a bias in the OOB error. The OOB error in more complex settings that include correlated predictors will be explored by means of real data.

### Real data-based studies

Based on the results from simulated data, real data sets were considered in which the overestimation of the OOB error is expected to be most pronounced. As will be seen later, a relevant bias of the OOB error is likely to occur in data settings with huge numbers of predictors, *p*, and small numbers of observations, *n*. Such settings are typically prevalent with genomic data. Therefore high-dimensional genomic data from the real world are considered for further investigations.

New data for evaluation can easily be generated with simulated data. In contrast to that, in real data applications, the original data has to be split up in order to obtain an independent test data set useable for evaluation. Thus, six genomic data sets were selected that are large enough to randomly split the data into a training and a test set (Table 3). These data sets were often used by various authors for classification purposes [16, 18, 19] and are briefly described in the following. Note that no pre-selection of data sets based on the results obtained for this data was performed, and the results of all six analyzed data sets are reported [20].

**Table 3:** Overview over high-dimensional genomic data sets.

Data set	No. response classes, *k*	No. pre-dictors, *p*	Considered *mtry* values	Size of original data
Colon Cancer	2	2000	{1, 10, 100, 500, 1000, 2000}	62
Breast Cancer	3	4869	{1, 10, 100, 500, 1000, 2000, 3000, 4000, 4869}	95
Breast Cancer	2	4869	{1, 10, 100, 500, 1000, 2000, 3000, 4000, 4869}	77
Prostate Cancer	2	6033	{1, 10, 100, 500, 1000, 2000, 3000, 4000, 5000, 6033}	102
Embryonal Tumor	2	7129	{1, 10, 100, 500, 1000, 2000, 3000, 4000, 5000, 6000, 7129}	60
Leukemia	2	7129	{1, 10, 100, 500, 1000, 2000, 3000, 4000, 5000, 6000, 7129}	72

#### Data

The first considered data is the *Colon Cancer data* of Alon et al. [21]. The expression levels of 40 tumor and 22 normal colon tissues for 6500 human genes were measured. The considered data set contains the expression of the 2000 genes with highest minimal intensity across the 62 tissues measured using the Affymetrix technology.

Two versions of the *Breast Cancer data* of van’t Veer et al. [22] were considered. The first version of this data was previously analyzed by Díaz-Uriarte and De Andres [16] and contains *k* = 3 response classes: 33 patients developed distant metastases within 5 years, 44 remained disease-free for over 5 years and 18 patients had *BRCA1* germline mutations. Missing data was imputed by using 5-nearest neighbor imputation. Further details on transformations of the original data are given in the supplement to the paper of Díaz-Uriarte and De Andres [16]. The second version which is considered in this paper is a subset of the data set provided by Díaz-Uriarte and De Andres [16]. This subset does not contain the 18 patients with *BRCA1* germline mutations. A differentiation is thus only made between the patients that developed distant metastases within 5 years (*n* = 33) and patients that remained disease-free for over 5 years (*n* = 44), meaning the number of response classes is *k* = 2.

The fourth considered data set is the *Prostate Cancer data* of Singh et al. [23]. From 1995 to 1997 samples of prostate tumors and adjacent non-tumor prostate tissue were collected from patients undergoing radical prostatectomy at the Brigham and Women’s Hospital. High-quality expression profiles were obtained from 50 non-tumor prostate samples and 52 tumor specimens. The oligonucleotide microarrays contained probes for approximately 12600 genes.

The *Embryonal Tumor data* of Pomeroy et al. [24] includes 60 patients with embryonal tumors of the central nervous system from whom biopsies were obtained before receiving treatment. The data was used to differentiate between patients who are alive after treatment (*n* = 21) and those who succumbed to their disease (*n* = 39) (data set C in [24]). RNA was extracted from frozen specimens and was analyzed with oligonucleotide microarrays containing 7129 probes from 6817 genes.

The *Leukemia data* [25] consists of 47 patients with acute lymphoblastic leukemia (ALL) and 25 patients with acute myeloid leukemia (AML). The considered data set comprises both, training samples and test samples from Golub et al. [25]. The samples were assayed using Affymetrix Hgu6800 chips and data on the expression of 7129 genes are available.

#### Settings

Different settings which were created by modifying the original real data sets were investigated. The aims together with the modifications are outlined in the following:

*Aim 1:* To quantitatively assess the overestimation in the OOB error and its consequences for selecting an optimal value for *mtry* using the OOB error. For this purpose, the original data was used without making any modifications to the data. This study is referred to as “Real data study”.
*Aim 2:* To investigate the behavior of the OOB error on data sets with realistic data structures but without any associations between the predictors and the response. To create a data set with realistic data structures, the matrix containing the values of the predictor variables of the real data sets was used and the response values of the original data sets were randomly permuted to break any associations between the predictors and the response. The studies with the permuted response are termed “Real data null case study with correlations”, where the term correlation refers to the correlations between the predictor variables. Note that the data sets obtained in this way only differ to the original data in that none of the predictors are associated with the response, while in the original data some of the predictors are possibly associated.
*Aim 3:* To investigate the effect of correlations on the bias in the OOB error in realistic data settings. For this purpose, each predictor variable was permuted separately to create independence between them. This also breaks possible associations between the predictors and the response. This setting is called “Real data null case study without correlations”. Note that, in order to assess the effect of correlations, the results for this study cannot be compared to the results obtained for the *real data study* (described above) because in the *real data study* some of the predictors are possibly associated with the response, while in the *real data null case study without correlations* this is not the case. This makes it impossible to decide whether differences are due to the correlations between predictors or are due to the fact that some of the predictors are associated in one study but not in the other. However, the results of the *real data null case study without correlations* can be compared to those of the *real data null case study with correlations*, in which there are correlations between predictors but none of the predictors is associated with the response.

Only a part of the observations was used to construct the RF (training set) while the other part was used for assessing the performance of the RF (test set). The number of trees was always set to 1000. For the data sets with *k* = 2 response classes, the training set consists of *n* = 20 observations that were randomly drawn, and for the Breast Cancer data (i.e. the only data set with *k* = 3), the training set consists of *n* = 30 observations. In contrast to the simulation studies, the response class ratio in the training set was not fixed. However, a minimum of 8 observations were required from each response class to prevent too few observations from a response class. With only *n* = 20 observations, this means that the response class distribution is nearly balanced and that only slight class imbalances can occur in the considered settings. Note that we chose to use only 20 and 30 observations, respectively, to train RF since these are settings in which a bias in the OOB error is most likely, as will be shown in the rest of this paper. Although modern studies include far more observations, such small sample sizes are still encountered in practice [26].

For all settings RF with different *mtry* values were constructed. The grid of *mtry* values was {1, 10, 100, 500, 1000, 2000, …, *p*}, with *p* denoting the total number of predictors. Table 3 shows the grids for the considered data sets. Each setting was repeated 1000 times.

### Alternative strategies for error estimation

The following strategies for error estimation were considered as possible alternatives to the OOB error:

- *Test error:* This error rate was computed using observations that are not part of the set of *n* observations that were considered for constructing the RF. Since these observations are usually referred to as test observations, the resulting error rate is referred to as test error. In the simulation studies, data for 10000 additional observations (test observations) was generated in order to estimate the prediction error of the RF. The response class distributions were the same in the two samples of size 10000 and *n*. In the real data studies, the *n* observations used to construct the RF (*n* = 20 for *k* = 2, *n* = 30 for *k* = 3) were randomly sampled from all available observations, while making sure that at least 8 observations from each response class were sampled. In order to have the same response class distribution in the two sets, as test set the largest subset of the remaining observations was used in which the response class distribution equals that in the sample of *n* observations.
- *Stratified OOB error:* In this paper, the OOB error was also computed for a RF based on a stratified sampling scheme. This strategy was also investigated in the studies of Mitchell [5]. In this stratified sampling scheme, trees were grown on subsamples of size ⌊0.632*n*⌋, in which the response class distribution of the original data of *n* observations is preserved in each subsample. The OOB error was computed based on the OOB observations as usual. In this paper, it is referred to as the stratified OOB error. Note that, in contrast to the test error, the (stratified) OOB error uses the *n* observations for both constructing the RF and estimating its prediction error.
- *Cross-validation (CV) error:* In contrast to the OOB error, CV is a strategy for estimating the error rate of an arbitrary classification method and is not specific to RF. In all studies 10-fold CV was used. Thus, for each constructed RF, the data was first partitioned into 10 sets of equal size. Each of the 10 sets was then used once for computing the error rate of the RF, while the other 9 sets were used for creating the RF. The CV error was computed as the average of the 10 error rates. While the test and OOB error (stratified and unstratified) estimation strategies use all of the *n* observations to construct the RF, in *l*-fold CV the *n* observations are split into a training and a test set and only the *n*(*l* − 1)/*l* training observations are used to construct a RF. This means that the CV error is computed from *l* models that are fit based on only a subset of the data. Thus, the CV error slightly overestimates the true prediction error that would be obtained for a model that was fit based on all *n* observations [27].
- *Stratified cross-validation (CV) error:* For computing the stratified CV error the data of size *n* was randomly split into *l* = 10 sets in a way, that within each set the distribution of response classes is the same as in the original data. The error estimation was then done in exactly the same way as was described for the CV error.

Since the test error is an accurate estimate for the generalization error, it is treated as a “gold standard” in this paper against which the OOB and CV errors (stratified and unstratified) are compared. In simulation studies, one should prefer estimating the error rate by means of an additional large independent test sample. In real data settings, in contrast, the number of observations is limited and is usually not sufficient to enable splitting the data into a training set and a large test set. Moreover, sample sizes are rather small and it is often desired to use all available information for building a model which has high predictive ability. Thus, in real data applications it is rarely the case that there is a large test set available from which the error rate can be computed (prior to externally validating the prediction model), and different approaches to estimating the error rate, such as cross-validation procedures, have to be applied.

### Random forest implementation and computational issues

The original RF version of Breiman and its implementation in the R package randomForest [12] was used for all studies. Note that stratified sampling will be investigated in the studies as possible solution to overcome the problem of the bias in the OOB error. In the RF implementation of Liaw and Wiener [12], one can specify a vector which contains the number of observations to be drawn from each class via the argument *sampsize* in the function randomForest. In the presence of categorical predictors, this RF version is biased with respect to split selection, because predictors with many possible cutpoints are preferentially selected [28–31]. This is, however, not a problem in the studies presented in this paper, because here exclusively metric predictors are considered. Nevertheless, to make sure that the results do not depend on the RF version considered, the RF version of Hothorn et al. [32] was used for some simulation settings in addition. This RF version, while computationally challenging, is unbiased with respect to split selection. Moreover, subsampling (i.e., sampling from the original data without replacement) was used in all studies instead of bootstrapping in order to avoid possible biases induced by the bootstrap [5, 29]. As was suggested, subsamples of size ⌊0.632*n*⌋ were used, where *n* denotes the number of observations [29]. Trees were always grown to full size. For this purpose default values for the parameters controlling tree size were not changed in the original RF version. In contrast to that, the parameters controlling tree size in the RF version of Hothorn et al. [32] were set to the most extreme values, such that early stopping was prevented.

## Results

**Fig 1:**
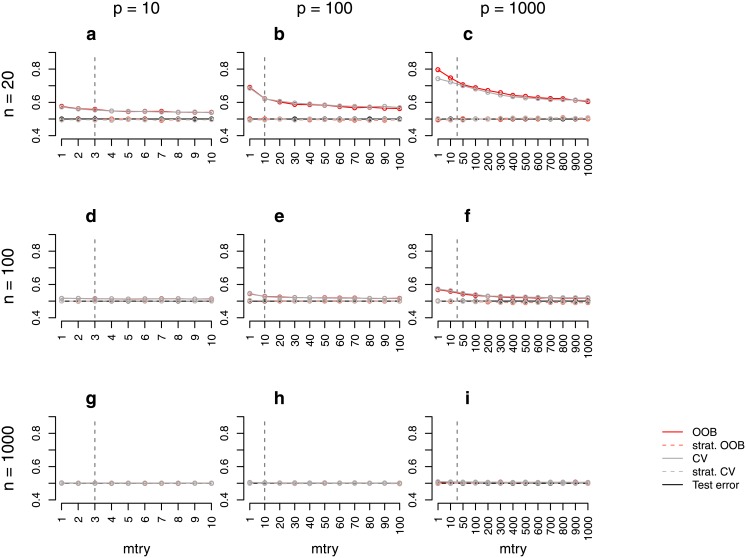
Error rate estimates for the *binary null case study (balanced)*. Shown are different error rate estimates for the setting with two response classes of equal size and without any predictors with effect. The error rate was estimated through the test error, the OOB error, the stratified OOB error, the CV error, and the stratified CV error for settings with different sample sizes, *n*, and numbers of predictors, *p*. The mean error rate over 500 repetitions was obtained for a range of *mtry* values. The vertical grey dashed line in each plot indicates the most commonly used default choice for *mtry* in classification tasks, that is $\left\lfloor \sqrt{p}\right\rfloor$.

**Fig 2:**
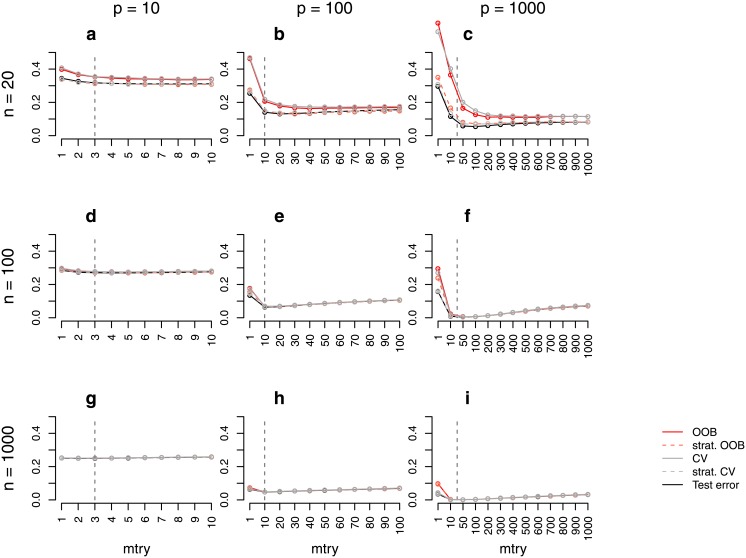
Error rate estimates for the *binary power case study (balanced)*. Shown are different error rate estimates for the setting with two response classes of equal size and with both predictors with effect and without effect. The error rate was estimated through the test error, the OOB error, the stratified OOB error, the CV error, and the stratified CV error for settings with different sample sizes, *n*, and numbers of predictors, *p*. The mean error rate over 500 repetitions was obtained for a range of *mtry* values. The vertical grey dashed line in each plot indicates the most commonly used default choice for *mtry* in classification tasks, that is $\left\lfloor \sqrt{p}\right\rfloor$.

**Fig 3:**
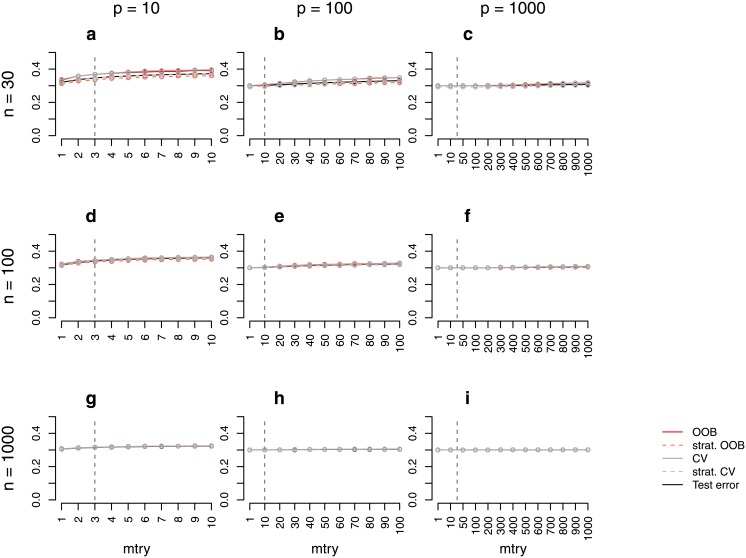
Error rate estimates for the *binary null case study (unbalanced)*. Shown are different error rate estimates for the setting with two response classes of unequal size (smaller class containing 30% of the observations) and without any predictors with effect. The error rate was estimated through the test error, the OOB error, the stratified OOB error, the CV error, and the stratified CV error for settings with different sample sizes, *n*, and numbers of predictors, *p*. The mean error rate over 500 repetitions was obtained for a range of *mtry* values. The vertical grey dashed line in each plot indicates the most commonly used default choice for *mtry* in classification tasks, that is $\left\lfloor \sqrt{p}\right\rfloor$.

**Fig 4:**
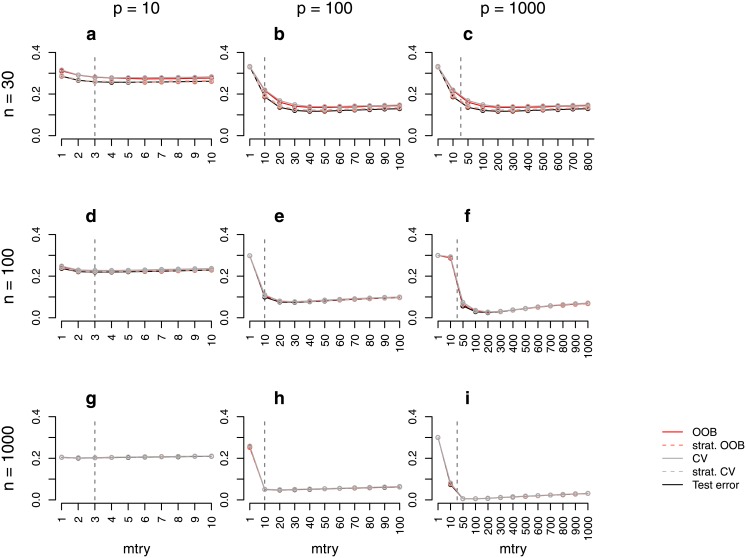
Error rate estimates for the *binary power case study (unbalanced)*. Shown are different error rate estimates for the setting with two response classes of unequal size (smaller class containing 30% of the observations) and with both predictors with effect and without effect. The error rate was estimated through the test error, the OOB error, the stratified OOB error, the CV error, and the stratified CV error for settings with different sample sizes, *n*, and numbers of predictors, *p*. The mean error rate over 500 repetitions was obtained for a range of *mtry* values. The vertical grey dashed line in each plot indicates the most commonly used default choice for *mtry* in classification tasks, that is $\left\lfloor \sqrt{p}\right\rfloor$.

**Fig 5:**
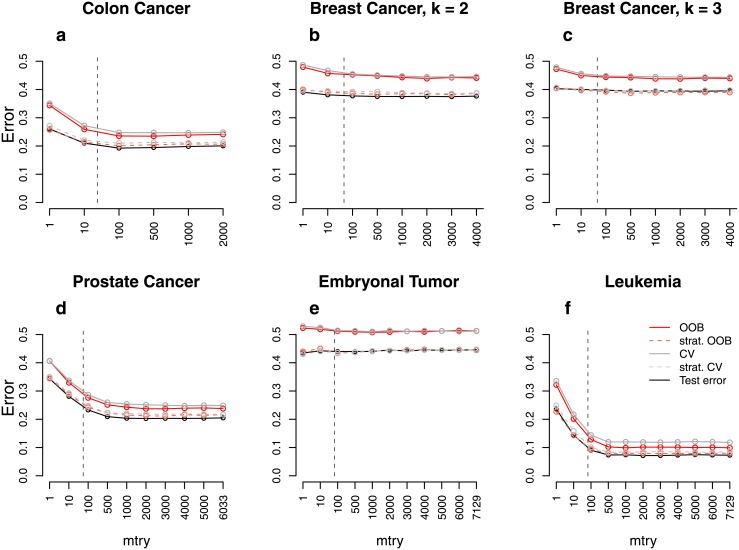
Error rate estimates for the *real data study*. Shown are different error rate estimates for six real data sets with two or three response classes, respectively, of nearly the same size. The error rate was estimated through the test error, the OOB error, the stratified OOB error, the CV error, and the stratified CV error for settings with different sample sizes, *n*, and numbers of predictors, *p*. The mean error rate over 1000 repetitions was obtained for a range of *mtry* values. The vertical grey dashed line in each plot indicates the most commonly used default choice for *mtry* in classification tasks, that is $\left\lfloor \sqrt{p}\right\rfloor$.

Figs 1–5 show the estimated error rates over a grid of *mtry* values for the five different error estimates (test error, OOB error, stratified OOB error, CV error, stratified CV error). In the following, the bias in the OOB error is quantified based on these results. Further the sources of the bias and the dependence of this bias on RF parameters and data characteristics are investigated, and finally the consequences of using the OOB error for tuning *mtry* are assessed.

### Quantitative assessment of the bias

For the *binary null case study (balanced)* the true error rate for new observations is 0.5, given that new observations come from both response classes equally often. Fig 1 shows the estimated error rates for the *binary null case study (balanced)*. The test error approximates 0.5 very well in all balanced settings and for all considered *mtry* values. For small sample sizes (Fig 1a, 1b and 1c; *n* = 20), the OOB error is larger than the test error which is considered to be a good estimate of the true prediction error. For larger sample sizes (Fig 1d, 1e and 1f; *n* = 100), the difference between the test error and the OOB error is smaller but still present. Finally, if the sample size is increased to *n* = 1000 (Fig 1g, 1h and 1i), the OOB error seems to approximate the test error well. When comparing the results for different parameter settings, it can be seen that the overestimation does not only depend on the number of observations but also on the number of predictors, or rather the ratio of the number of observations and predictors. In settings with both, large predictor numbers and small sample sizes (Fig 1c; *n* = 20, *p* = 1000), the overestimation is most extreme. Depending on the chosen value for *mtry*, the difference between the OOB error and the test error lies between 10% and 30% for this setting. In contrast, there is no overestimation in settings with large sample sizes and small predictor numbers (Fig 1g; *n* = 1000, *p* = 10). Exactly the same results are obtained for the CV error. This suggests that CV is not a reasonable alternative to the OOB error and, moreover, that there is a common source of the overestimation. In contrast to the OOB error and the CV error, the stratified OOB error and the stratified CV error approximate the test error very well and are reasonable alternatives to the unstratified sampling procedures in the considered study.

Comparable results were obtained for the *binary power case study (balanced)* (Fig 2). However, the difference between the OOB error (CV error) and the test error is smaller than in the study without any associations. In particular, there is only a small overestimation for large *mtry* values. Moreover, in contrast to the *binary null case study (balanced)*, there is no overestimation in settings with a moderate sample size of *n* = 100 (Fig 2d, 2e and 2f). Similar results were also obtained for balanced settings with four response classes (S1 File: Figs S1 and S2). This shows that the overestimation also occurs in settings with more than two response classes.

The findings of the *binary null case study (balanced)* and the *binary power case study (balanced)* do not transfer to the settings with unbalanced response classes. In the *binary null case study (unbalanced)*, the OOB error and the CV error are far closer to the test error (Fig 3). For the study with more extreme class imbalance (ratio 1:5) there are hardly any differences between the error rates estimated by the different strategies (S1 File: Fig S3). Overall, this suggests good performance of these two error estimation techniques in unbalanced data settings. It is not surprising that the prediction error is much lower than 0.5 in the unbalanced data settings. If for example all observations are classified into the larger class, one achieves an error rate which equals the proportion of the smaller class. With 30% observations belonging to the smaller class, the proportion of misclassified observations in a null case study could therefore be expected to be about 30%. These expectations are in line with the test error in Fig 3.

Some differences between the stratified OOB error and the test error can be observed in some of the power case settings (Figs 2c, 2f, 2i and 4c). In some balanced settings, the stratified OOB error is larger than the test error especially for *mtry* values close to one. However, such small *mtry* values are not recommended. In the presence of many variables without any effect, small *mtry* values prevent the selection of relevant variables yielding RF that have poor performance [17, 33].

**Fig 6:**
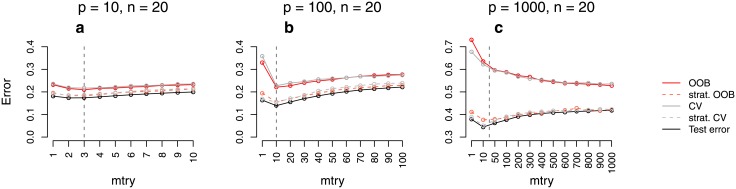
Error rate estimates for simulation studies with many predictors with effect and *n* = 20. Shown are different error rate estimates for an additional simulation study with two response classes of equal size and many predictor variables with effect. The error rate was estimated through the test error, the OOB error, the stratified OOB error, the CV error, and the stratified CV error for settings with sample size *n* = 20 and different numbers of predictors, *p*. The mean error rate over 500 repetitions was obtained for a range of *mtry* values. The vertical grey dashed line in each plot indicates the most commonly used default choice for *mtry* in classification tasks, that is $\left\lfloor \sqrt{p}\right\rfloor$.

Additional simulation studies with many predictor variables with effect show that if many predictors are associated with the response, there is a larger difference between the stratified procedures and the test error (Fig 6; see S1 File for details on the design). However, in all considered settings the difference between the stratified procedures and the test error is (substantially) smaller than that between the unstratified procedures and the test error.

To conclude, based on these results we have identified settings with (i) (nearly) balanced response classes, (ii) large predictor numbers, (iii) small sample sizes and (iv) a high signal-to-noise ratio as “high-risk settings” in which a large overestimation in the OOB error can be expected. By now the bias was quantified for rather simplistic settings which might not be realistic. The results for the real world high-dimensional genomic data sets in which (i)–(iv) apply, are shown in Fig 5. They are in line with the results obtained for the simulation studies: the OOB error and the CV error substantially overestimate the true prediction error for all data sets. The difference between the test error and the error estimated by the OOB procedure or CV is about 5%. CV performs worse than the OOB procedure for the Colon Cancer data, the Prostate Cancer data and the Leukemia data (Fig 5a, 5d and 5f). This might be related to the fact that the CV error is computed from models that are fit based on only a subset of the data, yielding only an upper bound of the prediction error [27]. Both CV and OOB error are very similar for the three remaining data sets. The stratified OOB error and the stratified CV error, in contrast, have a good performance approximating the test error very well. A marginal overestimation can, however, be seen for the stratified CV error and the stratified OOB error for two of the data sets (Colon Cancer data, Prostate Cancer data).

### Sources of the bias

The main source of the systematic deviation between the OOB error and the test error has already been described in the literature [5]. In the following, this main source is described before the bias and its dependence on specific parameters are detailed.

In a nutshell, the bias is attributable to the trees’ sensitivity to class imbalance. It is well known that classification trees are greatly affected by class imbalance in the sense that trees that were trained on unbalanced samples preferentially classify new observations into the class from which most training observations come. In the context of RF, where the classification trees are constructed using a subset of the data, this is also relevant to settings in which there is an equal number of observations from both classes. Later it will be shown that the impacts of this problem are even more severe for balanced than for unbalanced settings.

**Fig 7:**
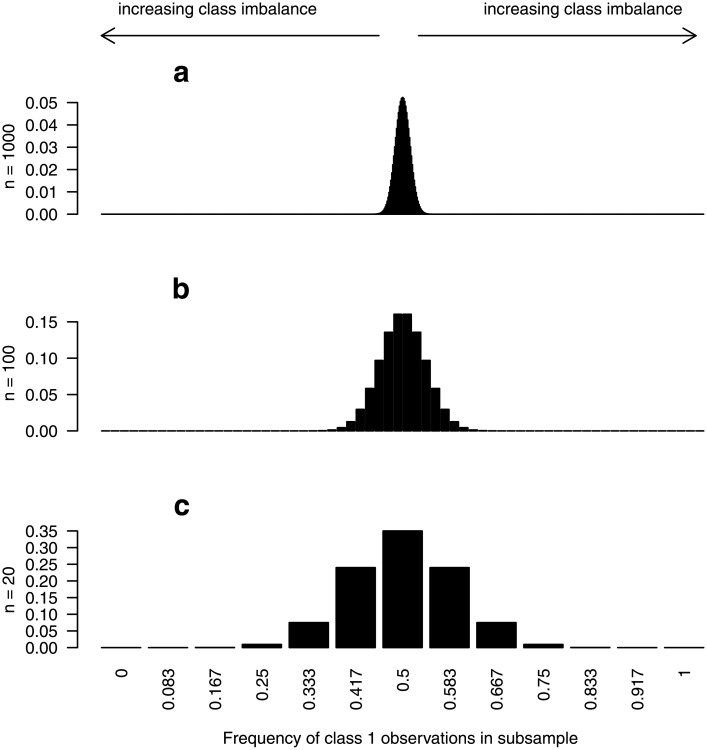
Class imbalance in subsamples drawn from a balanced original sample. Distribution of the frequency of class 1 observations in subsamples of size ⌊0.632*n*⌋, randomly drawn from a balanced sample with a total of (a) *n* = 1000, (b) *n* = 100, and (c) *n* = 20, observations from classes 1 and 2.

Let us assume in the following that we have a sample with an equal number of observations from both response classes. When constructing trees for a RF we randomly draw subsamples (or bootstrap samples) of observations from the original balanced sample. The subsample may comprise for example, 63.2% of the observations contained in the original sample. In contrast to the original sample, the resulting subsamples generally do not include exactly the same number of observations from each class, that is, the subsamples are often not exactly balanced or may even be extremely unbalanced if much more observations from one class are drawn by chance. The degree of class imbalance in the subsample is directly dependent on the sample size of the original sample, *n*. If *n* is large the chance for a stronger class imbalance in the subsample will be rather small, while for small *n*, the chance will be large. As an example, Fig 7 shows the degrees of class imbalance in subsamples of size 63.2% that are drawn from balanced samples of sizes *n* = 1000, *n* = 100 and *n* = 20. The distributions of the frequencies of class 1 observations in the subsamples were determined based on the hypergeometric distribution. As can be seen, there is a high chance of an extreme class imbalance for small samples. For large samples (*n* = 1000), in contrast, there is only a small degree of class imbalance. The class imbalance in the subsamples yields trees that preferentially predict the class most often represented in the subsample and the more extreme the class imbalance the more extreme the preferential prediction. Thus, the preferential prediction for a class is more pronounced for smaller samples than for larger samples.

If a prediction shall be obtained for a new observation, all trees in the RF are used to derive a prediction. Then we expect approximately the same number of trees preferentially predicting class 1 and trees preferentially predicting class 2. Overall, there is no preferential prediction for a new observation. In contrast to that, for OOB observations not all trees but only those trees for which the observation was not part of the subsample, are used to derive the prediction. If assuming that an observation *i* comes from class 1, for example, there are more subsamples without *i* that contain more observations from class 2 than subsamples without *i* that contain more observations from class 1. Accordingly, there are more trees for which observation *i* is “out-of-bag” preferentially predicting class 2, which is the wrong class. Again, the sample sizes play an important role. If the sample size is large, there are not substantially more subsamples without *i* that contain more observations from class 2. Then there is hardly any preferential prediction for the wrong class. In contrast to that, if the sample size is small, say *n* = 10, there are substantially more subsamples without *i* that contain more observations from class 2, yielding substantially more trees preferentially predicting the wrong class. The mechanism described above is the reason that the OOB predictions are worse than predictions that are obtained from the RF if the observation was not used for the construction of the RF. This mechanism finally leads to an OOB error that is too pessimistic, that is, it overestimates the error to be expected for new data.

In line with results from the literature, our studies suggest that a large amount of the overestimation can be solved by drawing subsamples in which the class distribution of the original data set is preserved [5]. All trees in the RF will then have the same preference for a class, and this preference will depend on the class distribution of the original sample. Thus, also the subset of the trees that is used to derive a prediction for an OOB observation have exactly the same preference for a class, which leads to OOB errors that are unbiased with respect to the error expected for independent test data. Note that computing the OOB error from an RF based on stratified subsamples with sampling fractions that are proportional to class sizes yields the stratified OOB error introduced in the section “Alternative strategies for error estimation”. The results shown in this paper support the findings of Mitchell [5] who claims that most of the bias can be eliminated by this alternative OOB error estimation.

In the following subsections, the reason for the dependence of the overestimation on data characteristics and RF parameters are investigated.

#### Role of the number of observations

The role of the sample size has already been described in detail. It was seen that large class imbalance in subsamples is especially a problem for smaller samples. The class imbalance results in trees that tend to more often predict the class that is more represented in the corresponding in-bag sample, or equivalently, that is less often represented in the corresponding OOB sample, leading to higher OOB errors. The dependence of the overestimation on the sample size is seen in the simulation results shown in the section “Quantitative assessment of the bias”. These show that the bias is almost negligible for *n* = 1000, while it is large for *n* = 20.

#### Role of *mtry*

Figs 1 and 2 show that, particularly for balanced data, the difference between the OOB error and the test error may strongly depend on the parameter *mtry*. While for balanced data the difference is larger for smaller *mtry* values (Figs 1 and 2), for unbalanced data this difference is, in contrast, smaller for smaller *mtry* values (Figs 3 and 4). The reasons for this are investigated separately for unbalanced and balanced settings in the following.

Let us first consider the setting with unbalanced data and no associations between the predictors and the response (*null case study*). Although there is no association between the predictors and the response in truth, some of the predictors may discriminate in-bag observations from different classes well by chance. If a large *mtry* value is used, these predictors are chosen for a split and the in-bag observations can be separated well. This yields trees that predict both classes and not only one of the classes (e.g. the most frequent class). In contrast to that, the well-discriminating predictors are not frequently selected as splitting variables in a tree if *mtry* is small. The resulting trees cannot discriminate between in-bag observations from different classes well and tend to predict the larger class more often. Then the RF, which uses the majority vote of the trees, predicts the larger class for almost all observations. This can also be seen by inspecting class predictions that are obtained from RFs with different *mtry* values in empirical studies. The inspection of class predictions was done using simulation studies and is outlined next.

**Fig 8:**
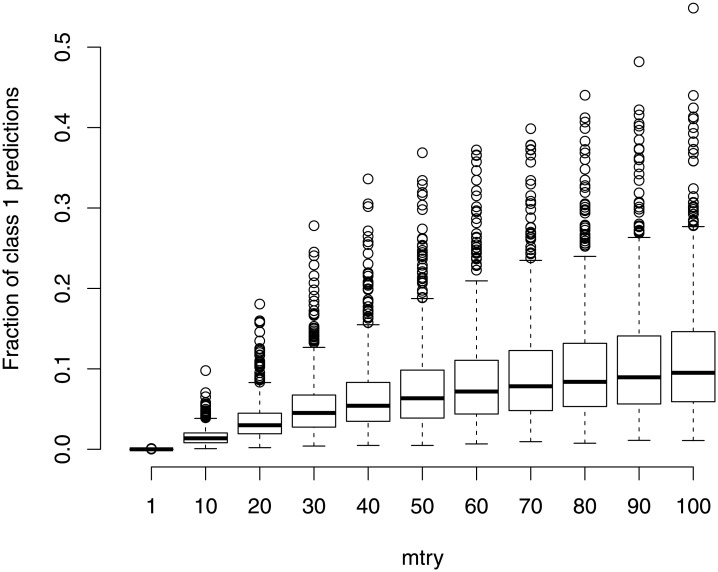
The trees’ preference for predicting the larger class in dependence on *mtry*. Fraction of class 1 (minority class in training sample) predictions obtained for balanced test samples with 5000 observations, each from class 1 and 2, and *p* = 100 (null case setting). Predictions were obtained by RFs with specific *mtry* (*x*-axis). RFs were trained on *n* = 30 observations (10 from class 1 and 20 from class 2) with *p* = 100. Results are shown for 500 repetitions.

Class predictions were obtained from RFs constructed using 10 observations from class 1 and 20 observations from class 2. The number of predictors, *p*, was 100. A null case scenario was simulated in which all predictors *X*_1_, …, *X*_100_ were drawn from a standard normal distribution. Predictions by the RFs were obtained for *n* = 10000 test observations, with an equal number of observations from class 1 and class 2. The proportion of class 1 (minority class) predictions for the test observations was finally computed. This process was repeated 500 times. Fig 8 shows the frequency of class 1 predictions over the 500 repetitions for different values of *mtry*. A clear trend can be seen that the larger class (class 2 in this simulation study) is more often predicted if *mtry* is small. For *mtry* values close to one, class 2 is almost always predicted.

The OOB error and the test error are almost the same if *mtry* is very small because most of the trees in the RF predict the larger class. In contrast to that, the trees do not always predict the larger class if *mtry* is large, and the phenomenon that for the OOB observations the trees tend to predict the opposite class becomes relevant again. This explains the finding that for large *mtry* the OOB error is more upwardly biased than for small *mtry*. However, in contrast to balanced settings in which the trees tend to predict the opposite class for an OOB observation, in unbalanced settings most of the trees have the preference for the same class, namely the largest class in the original sample. This reduces the risk that the trees tend to predict the opposite class for an OOB observation. Thus, the difference between the test error and the OOB error is far smaller in the unbalanced simulation settings than in the balanced simulation settings and is smallest in settings with very extreme class imbalance (S1 File: Fig S4).

Also note that, if *mtry* is set to 1 the prediction of only one class may yield low error rates in specific settings. These are settings in which most of the observations, for which the predictions shall be obtained, are from the class that is always predicted by the RF. For example, if the test data includes 30% of observations from class 1, and the RF always predicts class 2, then the test error is 30%. The same applies to the OOB error. In the simulated data, for example, the OOB error is estimated based on observations, in which approx. 70% of the observations come from class 2 and 30% come from class 1. In the case of small *mtry* values, the RF very frequently predicts class 2 (cf. Fig 8), yielding an OOB error close to 30%. This is also the reason why smaller test and OOB errors were obtained for smaller *mtry* values than for larger *mtry* values in the unbalanced null case scenarios, seen in Fig 3 and Fig S3 (S1 File). The other error estimation strategies are similarly affected.

Let us now consider the *balanced null case study*, in which there is an equal number of observations from all classes. When drawing samples for tree construction, it is usually the case that not exactly the same number of observations is drawn from each class. When drawing subsamples of size 0.632*n* from *n* = 20 observations (10 from each response class), for example, there is a 50% chance of obtaining subsamples with a different number of observations from each class (cf. Fig 7). When drawing from *n* = 100 observations, the chance to obtain an unbalanced subsample is about 84%. The trees grown on unbalanced samples tend to predict the larger class more often, especially if *mtry* is small. However, in contrast to the settings with an unbalanced original data, in the case of a balanced original sample there are approximately as many trees preferentially predicting class 1 as trees preferentially predicting class 2. In the absence of any associations between the predictors and the response, a new observation would then be classified to class 1 by 50% of the trees, while the other 50% of the trees classify the observation to class 2. This is independent of which value for *mtry* is chosen. Thus, there is no preferential prediction by the RF for new observations in balanced data settings. The test error computed from new observations is therefore not affected by different values for *mtry* if the original sample is balanced.

The OOB error, in contrast, is affected by the choice of *mtry* (cf. Fig 1). When obtaining predictions for an OOB observation *i* that comes from, say class 2, not all trees of a RF are used but only the trees that are constructed based on samples in which the observation was out-of-bag. Most importantly, even if the original sample is completely balanced, in the samples that do not contain the observation *i*, the proportion of observations from class 1 is higher on average than the proportion of observations from class 2. Thus, by construction, an OOB observation is out-of-bag for trees that tend to more often predict a class different than the true class the OOB observation belongs to. As explained before, this leads to the high OOB error rates observed in Fig 1. The OOB errors even exceed 0.5, which is the error rate of a random prediction in the absence of any associations between predictors and the response. As was outlined in the previous paragraph, the trees’ preference for the larger class in the subsample (i.e., most often the wrong class for the OOB observation) is stronger when small *mtry* values are used. This explains the finding that the OOB error is larger for RFs in which a small *mtry* value is used.

So far we focused on the case in which neither of the predictors are associated with the response. The mechanism described for the *null case study* may also play a role for the *power case study*, especially if there are only few predictors with effect and if the effects are small. In settings with only few influential predictors and many noise predictors, very small *mtry* values lead to trees that frequently select irrelevant variables for a split. Similar to the *null case study*, the trees then preferentially predict the class from which most training observations come. This explains the finding that in the simulation study (including only few relevant variables with rather small effects) the bias in the OOB error is larger for smaller *mtry* values in balanced settings, while the opposite is true for unbalanced settings.

#### Role of the predictors

**Fig 9:**
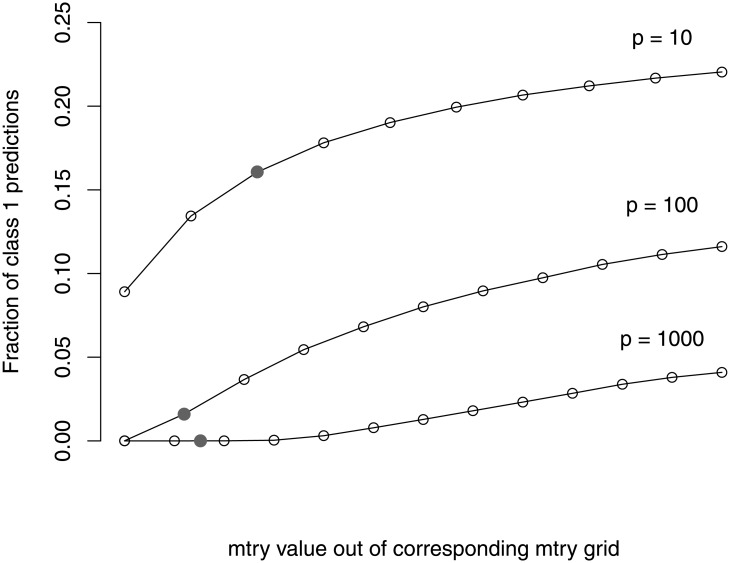
Trees’ preference for predicting larger class in dependence on *mtry* and number of predictors. Fraction of class 1 (minority class in training sample) predictions obtained for balanced test samples with 5000 observations from class 1 and 2, each (null case setting). Predictions were obtained by RFs with specific *mtry* from a corresponding grid of *mtry* values ({1, 2, …, 10} for *p* = 10, {1, 10, 20, …, 100} for *p* = 100, {1, 100, 200, …, 1000} for *p* = 1000). RFs were trained on *n* = 30 observations (10 from class 1 and 20 from class 2) with *p* ∈ {10, 100, 1000}. The mean fractions over 500 repetitions are shown. The grey dots indicate the most commonly used default choices for *mtry* in classification tasks, that is $\left\lfloor \sqrt{p}\right\rfloor$.

The simulation results have shown that the bias in the OOB error also greatly depends on the total number of predictors. This is again attributable to the trees’ preference for the larger class. It can be shown that the presence of more predictors leads to a more extreme preference for the majority class. The null case studies presented in the section “Role of *mtry*” and in Fig 8 were repeated for settings with *p* = 10 and *p* = 1000. Fig 9 shows the fraction of class 1 predictions (average of 500 repetitions) for *p* = 10, *p* = 100 and *p* = 1000. It shows that the preference for predicting class 1 by RF is more pronounced for settings with a larger number of predictors.

Again, depending on the class imbalance in the data used to construct the RF, a preference for the larger class can be of advantage or disadvantage for the bias in the OOB error. With unbalanced training data, a preference for the majority class will lead to a smaller bias in the OOB error; see the section “Role of *mtry*”. A larger bias in the OOB error will be obtained in contrast if the training data is balanced.

**Fig 10:**
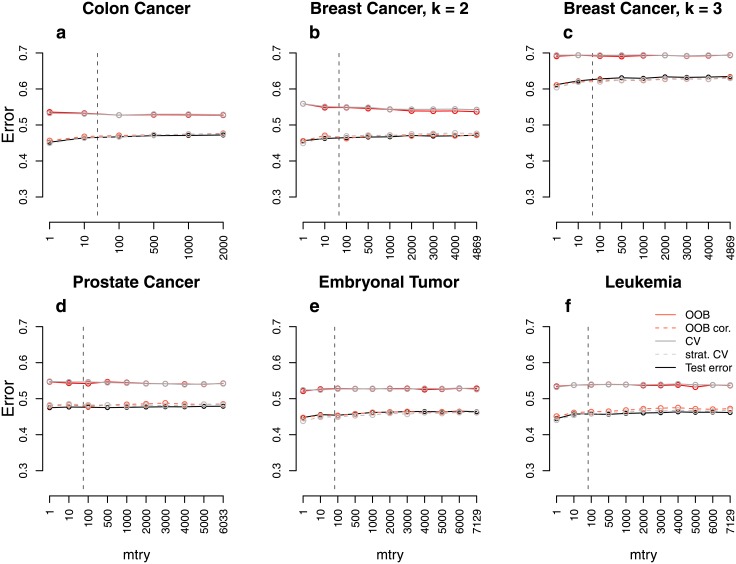
Error rate estimates for the *real data null case study with correlations*. Shown are different error rate estimates for studies based on six real data sets with correlated predictors and two or three response classes, respectively, of nearly the same size. The error rate was estimated through the test error, the OOB error, the stratified OOB error, the CV error, and the stratified CV error for settings with different sample sizes, *n*, and numbers of predictors, *p*. The mean error rate over 1000 repetitions was obtained for a range of *mtry* values. The vertical grey dashed line in each plot indicates the most commonly used default choice for *mtry* in classification tasks, that is $\left\lfloor \sqrt{p}\right\rfloor$.

**Fig 11:**
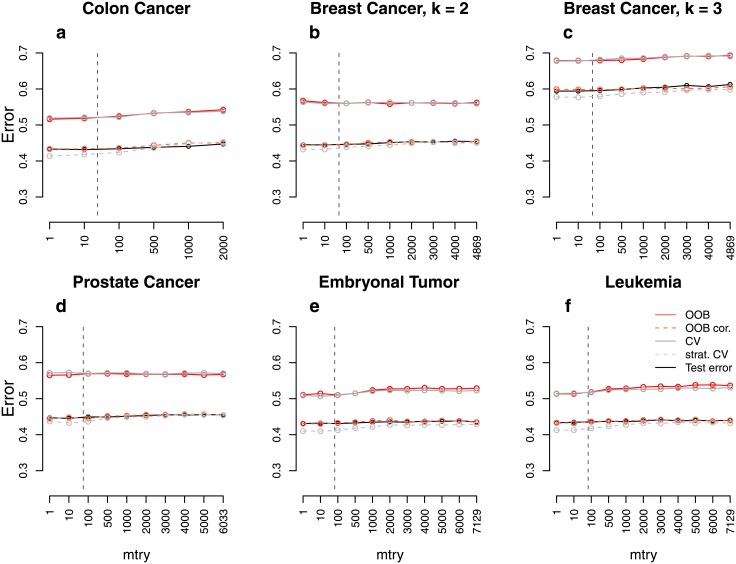
Error rate estimates for the *real data null case study without correlations*. Shown are different error rate estimates for studies based on six real data sets with uncorrelated predictors and two or three response classes, respectively, of nearly the same size. The error rate was estimated through the test error, the OOB error, the stratified OOB error, the CV error, and the stratified CV error for settings with different sample sizes, *n*, and numbers of predictors, *p*. The mean error rate over 1000 repetitions was obtained for a range of *mtry* values. The vertical grey dashed line in each plot indicates the most commonly used default choice for *mtry* in classification tasks, that is $\left\lfloor \sqrt{p}\right\rfloor$.

Correlations between predictors also play a role, as can be seen when comparing the results of the *real data null case studies* with and without any correlations, respectively (Figs 10 and 11). We observe that the bias of the OOB error and the CV error is larger if predictors are uncorrelated. Intuitively, if predictors are correlated, they contain more or less the same (or at least similar) information. Thus, there is less information contained in correlated predictors than in uncorrelated predictors. A similar mechanism occurs that has been described for the number of predictors: the less information that is contained in the data (e.g. due to a small number of predictors or high correlations), the less extreme the trees’ preference for one of the classes and the smaller the bias in the OOB error.

### Sampling the same number of observations per class in unbalanced data settings

RF’s preferential prediction of classes from which most training observations come from is a well-known phenomenon. A natural consequence of the preferential prediction is that new observations that in truth belong to the majority class have a high chance of correctly being classified into the larger class by the RF. In contrast to that, new observations from the minority class have only a small chance of correctly being classified into the smaller class. Therefore, it is sometimes of interest to measure the prediction performance of RF separately for observations from either class using the so-called class-specific OOB errors. For a class *j*, the class-specific OOB error is calculated analogous to the usual, class-unspecific OOB error, with the difference that not all training observations are considered, but only those from class *j*. The class-specific OOB errors as well as the class-specific test error were computed for the *binary extremely unbalanced* setting, and are represented by the solid lines in Fig S5 (S1 File; for the larger class) and Fig S6 (S1 File; for the smaller class). The class-specific OOB and test errors of the majority class are much smaller than those of the minority class, indicating a strong imbalance regarding the accuracy of RF for predicting observations from the different classes.

An approach called “balanced random forest” [34] tackles this imbalance by drawing the same numbers of observations with replacement from each class for each tree yielding trees that do not preferentially predict a specific class. This balanced RF approach is investigated in this section. The aim of these additional studies is to investigate the class-specific OOB error (and its bias) of the balanced RF approach and to compare it to the class-specific stratified OOB error with sampling fractions proportional to class sizes. In the original balanced RF approach, for each tree first a bootstrap sample is drawn from the smaller class and subsequently, a number of observations equal to the number of observations in the smaller class is drawn with replacement from the larger class. However, the problems associated with bootstrap in the case of standard RF can also be expected to occur for balanced RF. Therefore, the same numbers of observations from each class were drawn without replacement instead. The setting with the extremely unbalanced class sizes was used for this analysis. A number of n* = 0.75*n*_small_ observations were sampled without replacement from each class, where *n*_small_ denotes the number of observations from the smaller class. The number n* = 0.75*n*_small_ was used because using n* = 0.632*n*_small_ would result in very few training observations for each tree in cases in which the number of observations from the minority class is very small. The constant 0.75 was also recommended by Probst et al. [35], who used many publicly available data sets to find optimal default values for various tuning parameters and found that subsampling approximately 75% of observations for the trees in RF delivers good results in the majority of cases. The dashed lines in Figs S5 and S6 (S1 File) represent the class-specific OOB errors and the class-specific test error for the larger and the smaller class, respectively.

For both approaches (balanced RF and RF using stratified sampling with sampling fractions that are proportional to class sizes), the class-specific OOB errors can rarely be distinguished from the corresponding class-specific test errors. Thus, for both approaches, the class-specific OOB errors seem to be almost unbiased.

With respect to predictive ability we note that observations from the smaller class tend to be much better predicted for the balanced RF, in particular for smaller *mtry* values, including the commonly used choice $mtry=\lfloor \sqrt{p}\rfloor$. While observations from the larger class have more accurate predictions when performing stratified sampling with sampling fractions proportional to class sizes, the class-specific test errors of the larger class are also small when sampling the same numbers (except for the settings with *p* = 10). Note also that when sampling the same numbers, the class-specific test errors are almost identical for the two classes for each setting. This illustrates that sampling the same numbers of observations leads to an equal prediction performance for both classes.

For *p* = 10, the class-specific test errors of the larger class are quite high when sampling the same numbers and almost zero when using sampling fractions that are proportional to the class sizes. The reason for the former is that there is relatively few signal in the data for *p* = 10 (only two predictors with effect); the reason for the latter is that the larger class is almost always predicted when sampling fractions are proportional to class sizes. The latter preference for the larger class also explains why for *p* = 10 the overall (class-unspecific) test errors shown in Fig S7 (S1 File) are much lower using sampling fractions that are proportional to class sizes than when sampling the same numbers. As expected, no (relevant) systematic differences between the OOB errors and the corresponding overall test errors can be observed, both when sampling fractions are proportional to class sizes and sampling the same numbers. For *p* = 100, the overall test errors are similar between the two methods. This is also the case for *p* = 1000 if large values for *mtry* are chosen; if smaller *mtry* values are chosen, the overall OOB error obtained when sampling the same numbers is clearly smaller, in particular in the region of $mtry=\lfloor \sqrt{p}\rfloor$.

For unbalanced data, both when sampling the same numbers of observations (balanced RF) and when using sampling fractions that are proportional to the class sizes, the class-specific OOB errors are (almost) unbiased with respect to the corresponding class-specific test errors. Sampling the same numbers of observations from each class yields a RF that has the same prediction performance independent of the class an observation comes from. For observations from the smaller class prediction performance is considerably higher than that obtained when using sampling fractions that are proportional to the class sizes. Nevertheless, for observations from the larger class, sampling fractions that are proportional to the class sizes performs slightly better than sampling the same numbers from each class. In unbalanced settings, in which there is a strong interest in predicting observations from the smaller classes well, sampling the same number of observations from each class might therefore be the method of choice.

### Consequences for tuning *mtry*

The OOB error is frequently used to tune parameters like *mtry*. From the studies in the section “Quantitative assessment of the bias”, we have seen that the unstratified OOB error and the unstratified CV error often overestimate the true prediction error. Further, it was seen in some settings that the overestimation depends on *mtry*. This was not the case for the unstratified procedures, which were almost unbiased. In the following, the performance of RF when the *mtry* value is chosen based on the OOB error, the stratified OOB error, the CV error and the stratified CV error are compared. The performance was measured by the error rate which was computed based on an independent test data set. A different performance between RFs selected based on the stratified and the unstratified error estimation procedures would suggest that the bias affects tuning parameter selection, or in other words, that a suboptimal model might be chosen when the OOB error (or unstratified CV) is used for parameter tuning.

**Fig 12:**
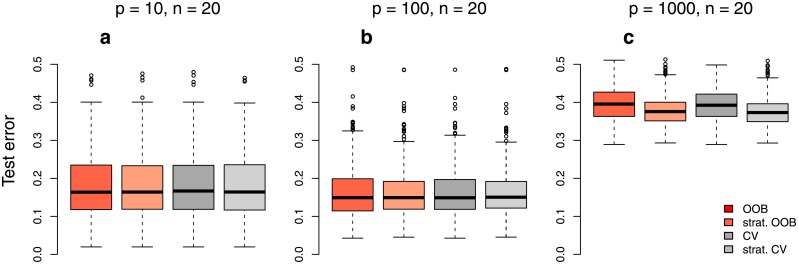
The effect of the bias of OOB error on RF’s performance when used for *mtry* selection. Performance of RF classifiers when *mtry* was selected based on the OOB error, the stratified OOB error, the unstratified CV error and the stratified CV error for the additional simulation studies with many variables with effect. The performance of RF was measured using a large independent test data set.

In the considered simulation studies and in the real data studies, there were no systematic differences between the error rates obtained when choosing *mtry* using the four methods (not shown). However, for the additional simulation studies with many variables with effect, there are differences in the settings with *p* = 1000 and *n* = 20. Fig 6c shows that a small *mtry* of 10 yields the RF with the best performance since the test error is smallest when using this *mtry* value. The OOB error, however, steadily decreases with larger values for *mtry*, suggesting that large values of *mtry*, such as 1000, should be used instead. Fig 12 shows the performance of the resulting RFs for 500 repetitions of the studies. For the setting with *p* = 1000 and *n* = 20 (Fig 12c) the mean difference in performance between the OOB error and the stratified OOB error is 1.5%, and the mean difference between the unstratified CV error and the stratified CV error is 1.9%. The bias in the OOB error thus impacts tuning parameter selection and leads to the selection of suboptimal classifiers in this case. However, the impact of the bias is very small and probably of no relevance in practice. For the two settings with smaller predictor numbers (*p* = 10, *p* = 100), there is again no difference between the four methods (Fig 12a and 12b).

A different concern arising in the context of using the OOB error for choosing the *mtry* value is whether using the OOB error both for choosing the *mtry* value and for error estimation leads to any (additional) bias of the OOB error as an estimate of the generalization error. Because the data is used twice in this scenario, first, for optimizing the *mtry* value and, second, for error estimation, this additional bias might be expected to lead to overoptimism, that is, too small error estimates. The following two procedures are associated with using the data twice, for choosing an optimal *mtry* value and for error estimation: (1) Use as error estimate of the RF the smallest OOB error obtained in the optimization, that is, the OOB error obtained for the chosen *mtry* value; (2) First, choose the *mtry* value using the OOB error, second, construct a RF using the *mtry* value chosen in the first step, third, calculate the OOB error of the latter RF and, lastly, use this OOB error as error estimate of the RF. In general, procedure (1) can be expected to be associated with a larger optimistic bias than procedure (2) because the smallest OOB error estimate obtained in the optimization can be expected to be overly small. For prediction methods different than RF, error estimation and tuning parameter optimization is usually performed using CV instead of OOB error estimation. In the latter context, the biases of procedures (1) and (2) have already been investigated in the literature, where it was confirmed that the bias of procedure (2) is much less severe than that of procedure (1) [36–38].

In order to investigate whether the biases of procedures (1) and (2) also apply to OOB error based optimization of the *mtry* value, a small simulation study was conducted in which only the *binary power case* setting with many variables with effect, *p* = 1000 and *n* = 20, was considered, again using 500 repetitions. This setting was chosen because the variability of the OOB error is largest for settings with small *n* and large *p*. In this analysis, procedures (1) and (2) were compared with respect to the test error, both when using stratified and unstratified subsampling. The results are shown in Fig S8 (S1 File). For both, stratified and unstratified subsampling, the errors estimated with procedure (1) are smaller than those estimated with procedure (2), confirming that the overoptimism from procedure (1) is larger than that of procedure (2). For unstratified subsampling, procedure (1) yielded error estimates that were slightly larger than the test error. Thus, in the considered setting the negative bias resulting from choosing the smallest OOB error estimate in procedure (1) was obviously strong enough to nearly neutralize the strong inherent positive bias of the unstratified subsampling described before. For stratified subsampling, for which we had observed no (relevant) bias when using fixed *mtry* values, there is a large downward bias of the error estimates from procedure (1), while the error estimates from procedure (2) are only slightly smaller than the test error.

Two main conclusions are drawn from these studies: (i) When choosing the *mtry* value using the OOB error, stratified subsampling can yield downwardly biased error rate estimates if the stratified OOB error that is smallest across all *mtry* values is used as an estimate of the generalization error; (ii) This bias can be greatly reduced by constructing a new RF using the *mtry* value that was chosen based on the stratified OOB error, and reporting the stratified OOB error of the new RF as an estimate of the generalization error. The latter point can be justified by the very small downward bias from procedure (2) that is observed for stratified subsampling in the analysis, even so the simulation setting with the highest variability of the OOB error estimates was used. Nevertheless, the gold standard procedure is using stratified CV for error estimation, choosing an optimal *mtry* value using the stratified OOB error in each iteration of the stratified CV.

## Discussion

Although it was shown that the OOB error may overestimate the true prediction error [2, 5], the OOB error is still often used in practice as an estimate of the true prediction error in classification tasks (see e.g. [39–41]).

The overestimation is due to the fact that—particularly in the case of balanced data sets—for a particular observation the in-bag samples used to predict the class of that observation tend to feature more observations from the other class. Given the trees’ preferential prediction of the class overrepresented in the training sample this leads to a tendency to predict the opposite class, which in turn leads to the observed overestimation of the error. Due to random variations, different response class distributions in the in-bag and the OOB samples are more likely when the original sample is small. This is the reason why in all the studies shown in this paper, the overestimation in the OOB error was large in small samples. This was also seen in the studies of Mitchell [5] who considered only a few, very specific settings with small sample sizes limiting the conclusions that could be drawn from those studies. The current studies not only confirm the result of Mitchell [5] that the OOB error is biased in balanced settings with small sample sizes, but they also show that there is hardly any overestimation in large samples, which is why the OOB error can be regarded as a good estimate of the true prediction error in large samples. Nevertheless, it is difficult to foresee in which settings the OOB error will be a good estimate of the true prediction error because there are many factors that affect the bias in the OOB error and there is an interplay between the factors. These factors are related to both the data and the parameters of RF.

Concerning parameters in RF, *mtry* was identified as parameter that has an influence on the bias of the OOB error. Additional studies were performed (not shown) that suggest that the parameters controlling the size of trees, in contrast, have no influence on the bias of the OOB error. Depending on the response class distribution in the original sample, larger values for *mtry* might increase (unbalanced settings) or decrease (balanced settings) the bias.

The influence of *mtry* on the bias in the OOB error might be problematic in the context of parameter tuning if the OOB error is used for selecting an appropriate value for *mtry*. However, although there was a clear influence of *mtry* on the bias in some of the studies, in only one of them this has lead to the selection of suboptimal RF classifiers. This can be explained by the fact that in nearly all studies, it seemed as if the specific choice of *mtry* was not crucial. There was a wide range of *mtry* values that yielded optimal performance, especially for the high-dimensional genomic data sets with values for *mtry* larger than 100 yielding very similar performance. However, one cannot be sure that this applies to all future data sets. Among our studies there was one study with a clear performance peak at a specific *mtry* value. In this setting the tuning parameter selection based on the stratified OOB error yielded slightly more accurate RF models than that based on the classical, that is the unstratified, OOB error.

With respect to data-dependent factors, the present studies identified the response class distribution of the original sample, the predictor number, the correlation between predictors as well as their predictive ability as relevant factors that have an effect on the bias. The studies reported in the literature consider only settings in which there is an equal number of observations from all response classes [5]. The results in this paper show that the effect of *mtry* on the bias depends on the response class distribution of the original sample. For completely balanced samples, we observed a more extreme overestimation of the true error rate for smaller values of *mtry*. For unbalanced samples the opposite was true. This again underlines that it is difficult to assess whether there will be any bias in future real data applications and how severe this bias is because it depends on several different factors acting together.

In the context of obtaining powerful RF prediction models in the presence of unbalanced data, it might be worthwhile to consider sampling the same number of observations from each response class—in particular, if there is a strong interest in obtaining valid predictions for observations coming from the smaller class as well. This approach was also investigated in this paper for settings with extreme class imbalance and class-specific and overall error rates were assessed. The results suggest that sampling the same numbers of observations from each class could be a promising alternative to using sampling fractions that are proportional to class sizes, since it yielded unbiased OOB error estimates in the considered settings as well.

Of note, the problem that leads to the overestimation in the error rate is not specific to OOB estimation in RF, but is relevant to any data splitting procedure, such as cross-validation. These procedures can be expected to deliver upwardly biased error estimates for any classification method that is sensitive towards class imbalance, which is why many of the results apply to other methods beyond RF as well. In the present studies 10-fold cross-validation also yielded too pessimistic error rates. Therefore, cross-validation and related procedures are not alternatives for preventing the overestimation. Instead stratified procedures, such as stratified cross-validation, have been recommended to bypass this problem [42]. The use of stratified cross-validation for error estimation in the context of RF has not been systematically investigated so far. In the present studies, stratified cross-validation resulted in good approximations of the true prediction error of RF in the considered settings.

It should also be noted that error estimates based on data splitting procedures, such as (stratified) cross-validation estimates or OOB estimates, are, in general, associated with a high variance [43]. There are, however, no general alternative approaches for estimating the generalization error of a prediction model using a single training data set. A more precise error estimate that is in many cases also more realistic can be obtained using a large external validation data set. Note that before applying a prediction model in practice, external validation should always be performed [44, 45].

In benchmarking studies, cross-validation is often applied to compare the performance of different statistical methods. If it is applied in a non-stratified manner, it might happen that the performance for RF might appear worse than it actually is. If RF (or a different method that is sensitive towards class imbalance) is considered as a competing method in a benchmark study, it is recommended to use stratified cross-validation to avoid misinterpretations on the performance of RF or other methods that are similarly affected. Note that this problem is relevant especially to settings in which the original data contains (almost) exactly the same number of observations from the response classes, that is, it is *not* a problem that is encountered especially in unbalanced data settings.

In the original RF version of Breiman [1], the trees are constructed based on bootstrap samples. In the studies of Mitchell [5], the use of bootstrap sampling was shown to further increase the bias. Irrespective of this, bootstrap sampling has been shown to induce a preferential selection of certain types of predictors for a split [29]. Therefore, the use of bootstrapping in RF is disapproved to avoid misleading conclusions, and the R package party, for example, draws subsamples by default for this reason. Accordingly, the results in this paper are shown for RF that are always constructed based on subsampling—either unstratified or stratified, the latter leading to the correction addressed above.

The studies shown in this paper are mainly based on the original RF version of Breiman [1]. Some of the simulation settings were also performed with the RF version based on conditional inference trees [32] implemented in the R package party to assess whether there are any differences (results not shown). The results obtained for this RF version were very similar suggesting that the conclusions drawn from the studies are not specific to the RF version used. Moreover, the problem is not specific to the use of the error rate as performance measure. Any different measure is affected in the same manner. The area under the curve (AUC), for example, represents the probability that for an observation from the diseased class the predicted probability of being diseased is higher than for an observation from the class of healthy subjects [46]. It is often used as an alternative to the error rate for assessing the prediction accuracy in unbalanced binary classification settings. However, the AUC computed from OOB observations similarly underestimates the true AUC, and one cannot circumvent the problem of the biased OOB error by using a performance measure different than the error rate.

Both the stratified OOB error and the error rate computed from stratified cross-validation also overestimated the true prediction error in some of our studies with metric predictor variables. The overestimation was larger if many variables were associated with the response and only marginal if only few variables were associated. Overall, the overestimation through the stratified procedures was considerably smaller than that obtained through the unstratified procedures, supporting the use of stratified procedures. Future studies might aim at developing alternative error estimation strategies that are both unbiased and computationally tractable.

Finally note that the example data sets considered in our studies are all from the medical field. RF is, however, used in a large variety of application areas and the results and recommendations given in the paper are not limited to applications in the medical field.

## Conclusions

Prior to our work, little had been known about the bias of the OOB error, and the OOB error is still frequently used for error estimation in classification settings. Using simulation-based and real-data based studies with metric predictor variables, it was shown that the overestimation is not restricted to binary classification settings and that it is largest in settings with

- an equal number of observations from all response classes (i.e., balanced samples),
- small sample sizes,
- a large number of predictor variables,
- small correlations between predictors and
- weak effects.

These factors act together making it difficult to foresee in which settings the OOB error will greatly overestimate the true prediction error.

The overestimation encountered in settings with metric predictor variables can depend on the parameter *mtry*. This might be a problem when the OOB error is used for selecting an appropriate value for *mtry*, a procedure frequently performed in practice. Overall, however, the prediction performance of RF was not substantially affected when using the OOB error for selecting an appropriate value for *mtry* in the studies shown in this paper. However, one cannot be sure that this applies to all future data.

In line with results reported in the literature [5], the use of stratified subsampling with sampling fractions that are proportional to response class sizes of the training data yielded almost unbiased error rates in most settings with metric predictors. It therefore presents an easy way of reducing the bias in the OOB error. It does not increase the cost of constructing the RF, since unstratified sampling (bootstrap of subsampling) is simply replaced by stratified subsampling.

For any settings that include only metric predictor variables it is thus strongly recommended to use stratified subsampling with sampling fractions that are proportional to class sizes in place of unstratified sampling that is, by default, used in RF. This reduces the risk for misinterpretations regarding the predictive accuracy of RF, and might avoid choosing a value for *mtry* that possibly leads to suboptimal performance when using the OOB error for parameter tuning.

## Supporting information

S1 File PDF file containing Supplementary Information. Various contents referred to in the paper.
(PDF)
