# Supplementary material for: On the overestimation of random forest’s out-of-bag error
Source: PLoS One. 2018 Aug 6;13(8):e0201904. doi: 10.1371/journal.pone.0201904 (PMC6078316; doi:10.1371/journal.pone.0201904)
Supplement: S1 File — Various contents referred to in the paper. (PDF) [file pone.0201904.s001.pdf]

Supplementary Information to the PLOS ONE article:

On the overestimation of random forest's out-of-bag error

Silke Janitza*[,1] and Roman Hornung[1]

[1] Institute for Medical Information Processing, Biometry and Epidemiology, University of Munich, Munich, 81377, Germany

*To whom correspondence should be addressed: silke.janitza@gmail.com

A Results of multiclass power and null case studies

The results of the *multiclass null case study* and the *multiclass power case study* are shown in Figs S1 and S2.

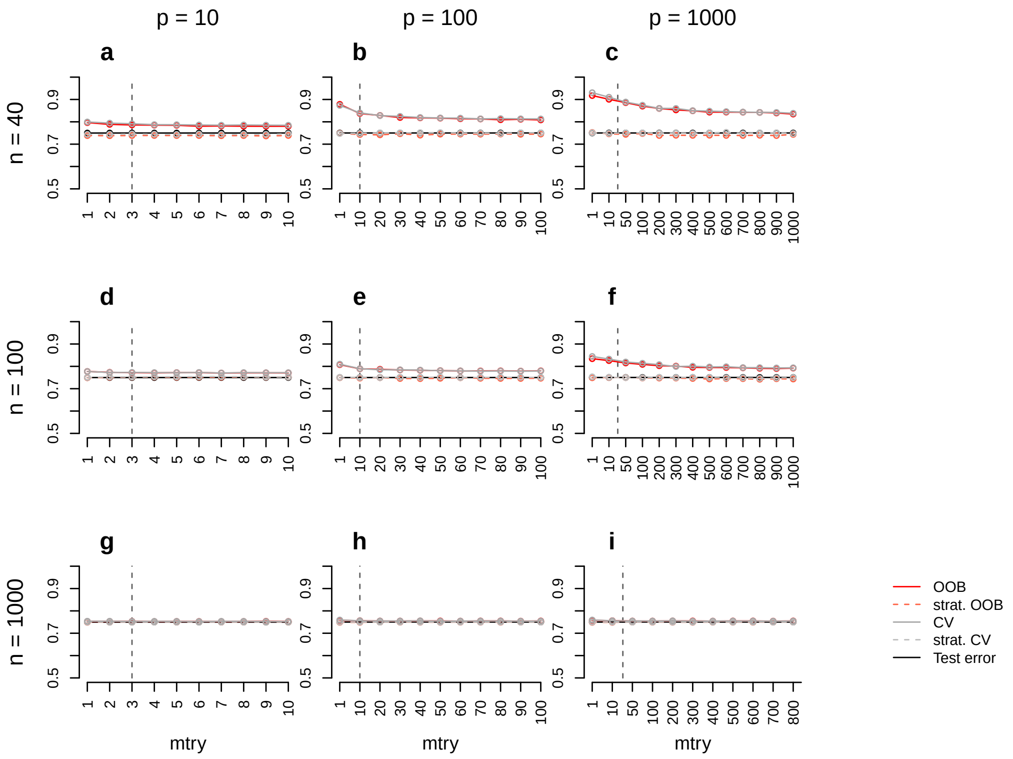

Fig. S1: **Error rate estimates for the *multiclass null case study (balanced)*.** Shown are different error rate estimates for the setting with four response classes of equal size and predictors without any effect. The error rate was estimated through the test error, the OOB error, the stratified OOB error, the CV error, and the stratified CV error for settings with different sample sizes, n, and numbers of predictors, p. The mean error rate over 500 repetitions was obtained for a range of *mtry* values. The vertical grey dashed line in each plot indicates the most commonly used default choice for *mtry* in classification tasks, that is $\lfloor\sqrt{p}\rfloor$.

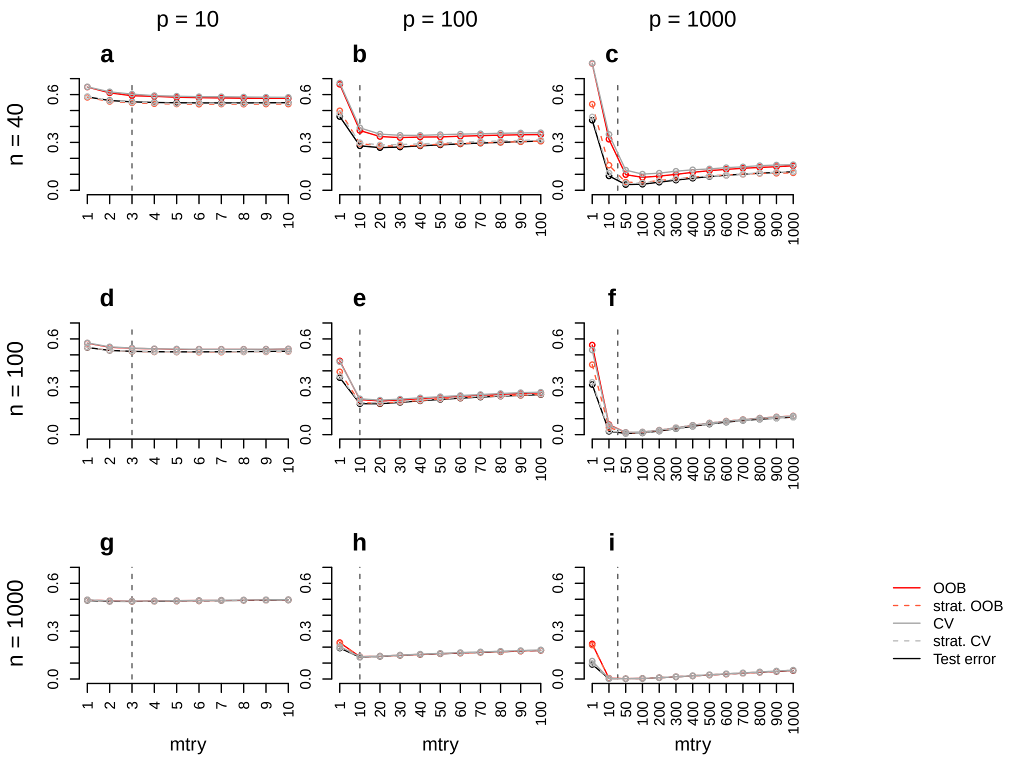

Fig. S2: **Error rate estimates for the *multiclass power case study (balanced)*.** Shown are different error rate estimates for the setting with four response classes of equal size and both predictors with effect and without any effect. The error rate was estimated through the test error, the OOB error, the stratified OOB error, the CV error, and the stratified CV error for settings with different sample sizes, n, and numbers of predictors, p. The mean error rate over 500 repetitions was obtained for a range of *mtry* values. The vertical grey dashed line in each plot indicates the most commonly used default choice for *mtry* in classification tasks, that is $\lfloor\sqrt{p}\rfloor$.

B Results of binary power and null case studies with extreme class imbalance (ratio 1:5)

The results of the study with binary response and class imbalance ratio 1:5 are shown in Figs S3 and S4.

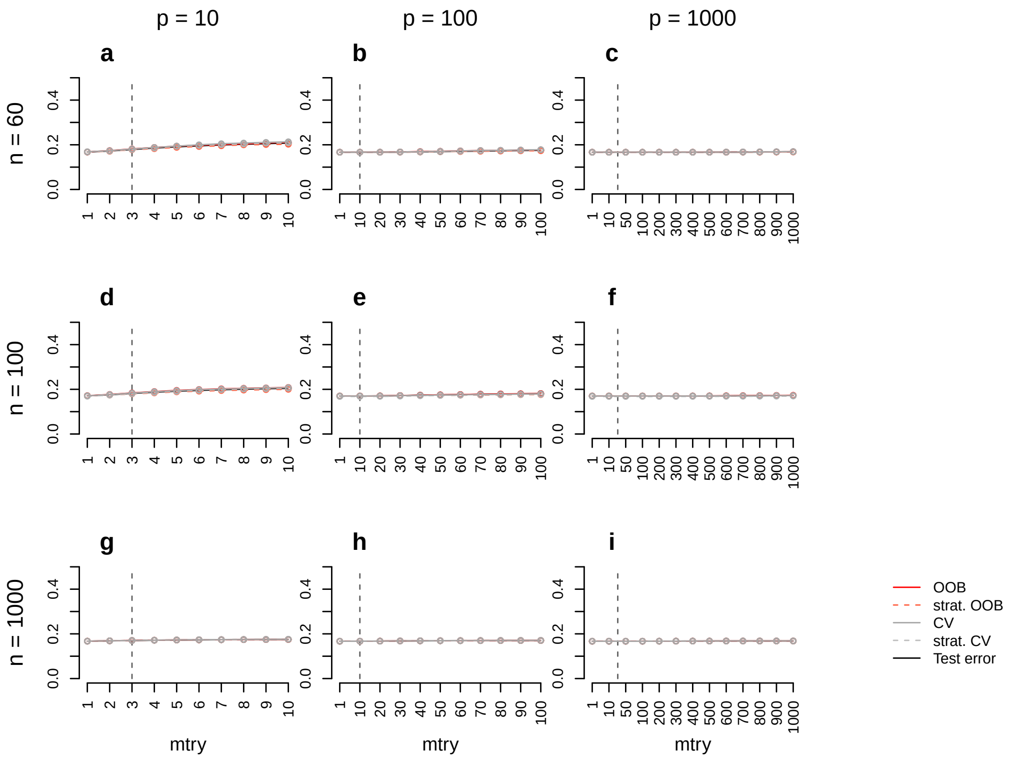

Fig. S3: **Error rate estimates for the *binary null case study (extremely unbalanced)*.** Shown are different error rate estimates for the setting with two extremely unbalanced response classes (ratio 5:1) and predictors without any effect. The error rate was estimated through the test error, the OOB error, the stratified OOB error, the CV error, and the stratified CV error for settings with different sample sizes, n, and numbers of predictors, p. The mean error rate over 500 repetitions was obtained for a range of *mtry* values. The vertical grey dashed line in each plot indicates the most commonly used default choice for *mtry* in classification tasks, that is $\lfloor \sqrt{p} \rfloor$.

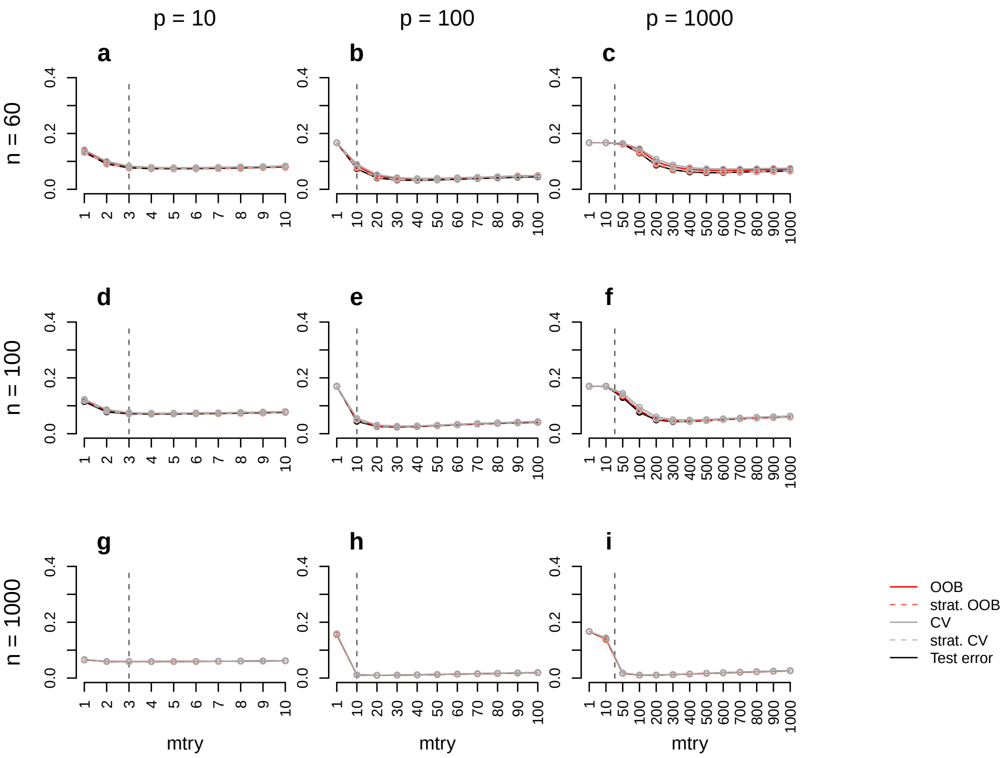

Fig. S4: **Error rate estimates for the *binary power case study (extremely unbalanced)*.** Shown are different error rate estimates for the setting with two extremely unbalanced response classes (ratio 1:5) and both predictors with effect and without any effect. The error rate was estimated through the test error, the OOB error, the stratified OOB error, the CV error, and the stratified CV error for settings with different sample sizes, n, and numbers of predictors, p. The mean error rate over 500 repetitions was obtained for a range of *mtry* values. The vertical grey dashed line in each plot indicates the most commonly used default choice for *mtry* in classification tasks, that is $\lfloor\sqrt{p}\rfloor$.

C Additional simulation studies: Binary power case study with many predictors with effect

A study with many predictors with effect was simulated. The predictors not associated with the response followed a standard normal distribution. The distribution of predictors with association was different for the two response classes. The predictor values for observations from class 1 were always drawn from a standard normal distribution. The predictor values for observations from class 2 were drawn from a normal distribution with variance 1 and a mean that was in turn drawn from a normal distribution specified in Table S1.

Table S1: **Simulation design of simulation study with many predictors with effect**

No. predictors	Predictors	Class 1<br>$N(\mu_1, 1)$	Class 2<br>$N(\mu_2, 1)$
$p = 10$	X_1	$\mu_1 = 0$	$\mu_2 \sim N(0, 1)$
	$\vdots$	$\vdots$	$\vdots$
	X_8	$\mu_1 = 0$	$\mu_2 \sim N(0, 1)$
	X_9, X_{10}	$\mu_1 = 0$	$\mu_2 = 0$
$p = 100$	X_1	$\mu_1 = 0$	$\mu_2 \sim N(0, 0.6^2)$
	$\vdots$	$\vdots$	$\vdots$
	X_{50}	$\mu_1 = 0$	$\mu_2 \sim N(0, 0.6^2)$
	$X_{51}, \ldots, X_{100}$	$\mu_1 = 0$	$\mu_2 = 0$
$p = 1000$	X_1	$\mu_1 = 0$	$\mu_2 \sim N(0, 0.2^2)$
	$\vdots$	$\vdots$	$\vdots$
	X_{500}	$\mu_1 = 0$	$\mu_2 \sim N(0, 0.2^2)$
	$X_{501}, \ldots, X_{1000}$	$\mu_1 = 0$	$\mu_2 = 0$

Distribution of predictors in class 1 and class 2 in the binary power case study with many predictors with effect.

D Results of study on class-specific OOB errors and on sampling the same numbers of observations per class in unbalanced data settings

The results of the study on class-specific OOB errors and on sampling the same numbers of observations per class are shown in Figs S5, S6 and S7.

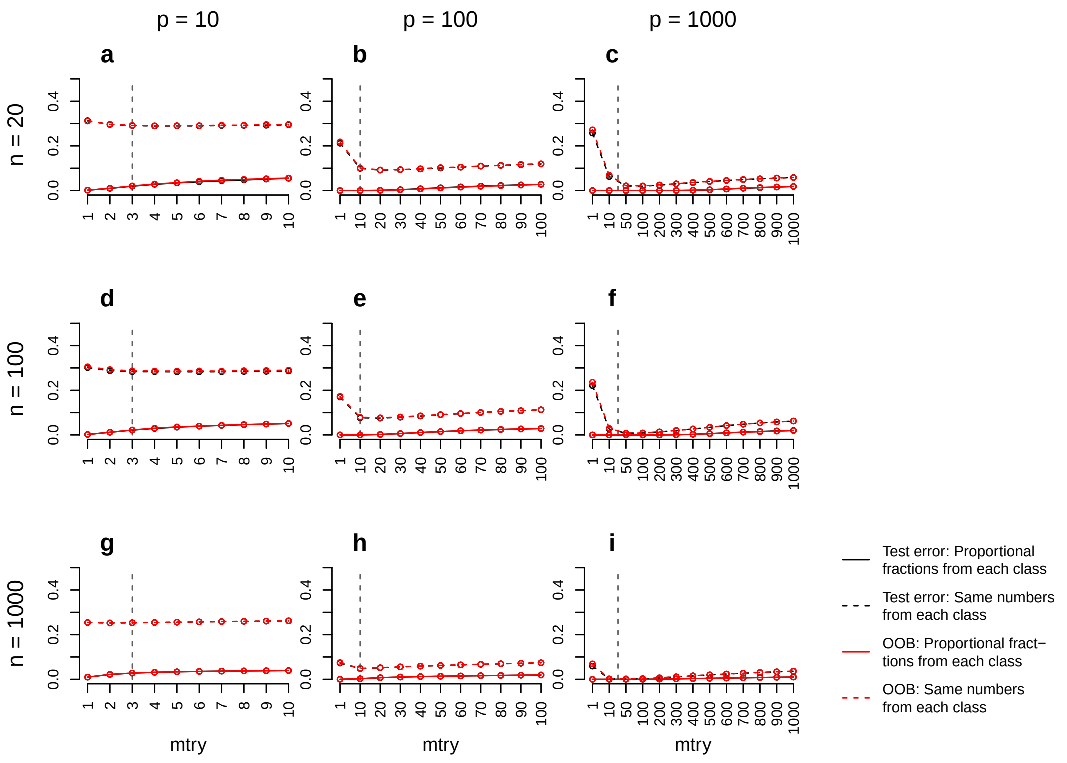

Fig. S5: **Class-specific error rate estimates of the larger class for the study on class-specific OOB errors and on sampling the same numbers of observations per class.** Shown are the mean test error estimates obtained for all test set observations from the larger class when using sampling fractions that are proportional to the class sizes and when sampling the same numbers of observations from each class for the setting with two extremely unbalanced response classes (ratio 5:1) and both predictors with effect and without any effect. The mean error rate over 500 repetitions was obtained for a range of *mtry* values. The vertical grey dashed line in each plot indicates the most commonly used default choice for *mtry* in classification tasks, that is $\lfloor\sqrt{p}\rfloor$.

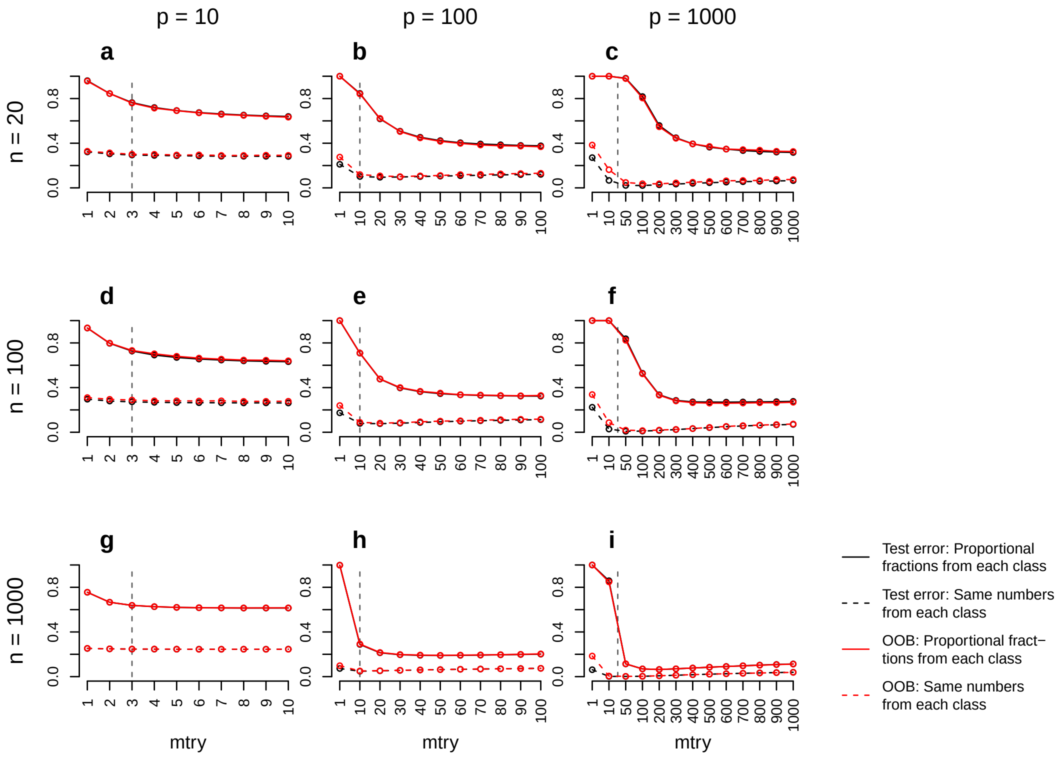

Fig. S6: **Class-specific error rate estimates of the smaller class for the study on class-specific OOB errors and on sampling the same numbers of observations per class.** Shown are the mean test error estimates obtained for all test set observations from the smaller class when using sampling fractions that are proportional to the class sizes and when sampling the same numbers of observations from each class for the setting with two extremely unbalanced response classes (ratio 5:1) and both predictors with effect and without any effect. The mean error rate over 500 repetitions was obtained for a range of *mtry* values. The vertical grey dashed line in each plot indicates the most commonly used default choice for *mtry* in classification tasks, that is $\lfloor\sqrt{p}\rfloor$.

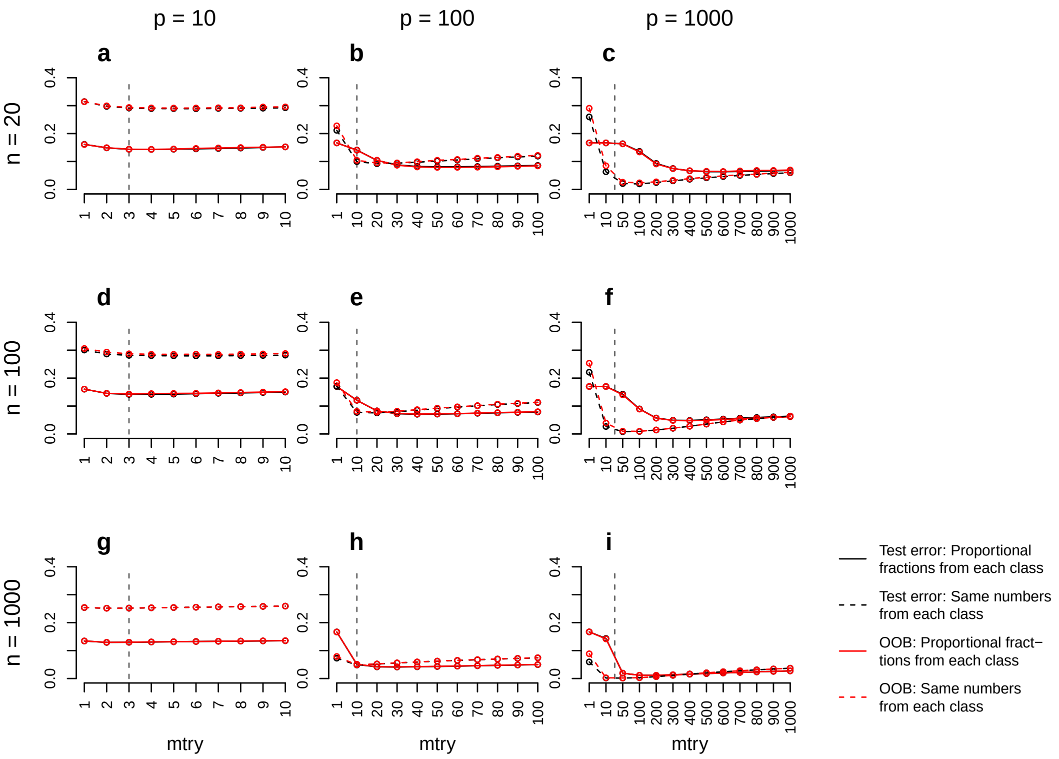

Fig. S7: **Error rate estimates for the study on class-specific OOB errors and on sampling the same numbers of observations per class.** Shown are the mean test error estimates obtained for all test set observations when using sampling fractions that are proportional to the class sizes and when sampling the same numbers of observations from each class for the setting with two extremely unbalanced response classes (ratio 5:1) and both predictors with effect and without any effect. The mean error rate over 500 repetitions was obtained for a range of *mtry* values. The vertical grey dashed line in each plot indicates the most commonly used default choice for *mtry* in classification tasks, that is $\lfloor\sqrt{p}\rfloor$.

E Results of study on using the OOB error both for tuning and error estimation

The results of the study on using the OOB error both for tuning of *mtry* and error estimation are shown in Fig S8.

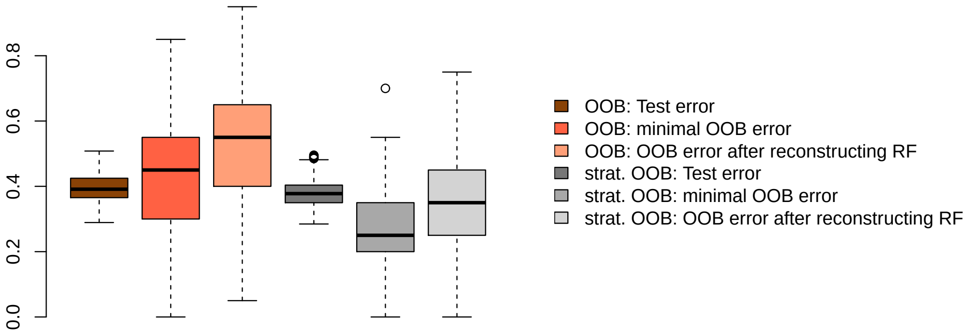

Fig. S8: **Error rate estimates for the study on using the OOB error both for tuning of *mtry* and error estimation.** Shown are different error rate estimates for the setting with many variables with effect, $p = 1000$ and $n = 20$. The error rate was estimated through the test error obtained after optimization using the OOB error estimate, the lowest OOB error estimate obtained in the optimization, the OOB error estimate of an RF constructed after optimization using the OOB error estimate, the test error obtained after optimization using the stratified OOB error estimate, the lowest stratified OOB error estimate obtained in the optimization, and the stratified OOB error estimate of an RF constructed after optimization using the stratified OOB error estimate. The boxplots show the values obtained in 500 repetitions.
